# Role of CSF flow and meningeal barriers in the development of inflammatory lesions at the CNS–PNS transition zone of cranial nerves in autoimmune demyelinating diseases

**DOI:** 10.1007/s00401-025-02896-1

**Published:** 2025-06-19

**Authors:** Li Xin, Hideaki Nishihara, Adrian Madarasz, Petr Pleskac, Linh Tran, Daniela C. Ivan, Fumitaka Shimizu, Simone Aleandri, Giuseppe Locatelli, Paola Luciani, Steven T. Proulx

**Affiliations:** 1 Theodor Kocher Institute, University of Bern, CH-3012 Bern, Switzerland; 2 Department of Neurology and Clinical Neuroscience, Yamaguchi University, Yamaguchi, Japan; 3 Department of Chemistry, Biochemistry and Pharmaceutical Sciences, University of Bern, Bern, Switzerland

**Keywords:** Cerebrospinal fluid (CSF), Cranial nerve lesions, CCR2^+^ cells, CNS–PNS transition zone, Neuroinflammation

## Abstract

Patients with autoimmune inflammatory demyelinating diseases have been shown to present with trigeminal and cochlear nerve lesions restricted at the root transition zone, which contrasts with the relatively extensive distribution of lesions in optic neuritis. To better understand the mechanism underlying the different distribution pattern for cranial nerve lesions in these autoimmune neuroinflammatory diseases, we focused on the CNS–PNS transition zone (TZ) of the trigeminal and cochlear nerves in a MOG-driven active EAE model. These nerves were found to exhibit unique arrangements of anatomical barrier layers including the arachnoid and glia limitans, which affected cerebrospinal fluid (CSF) tracer distribution as well as CCR2^+^ immune cell infiltration. Our data demonstrated that CCR2^+^ immune cells accumulate at the TZ on both CNS side and PNS side of the trigeminal nerve and cochlear nerve, which mirror the locations of cranial nerve pathology observed clinically in patients with inflammatory demyelinating disease. On the other hand, the optic and olfactory nerves, which both lack a TZ, did not exhibit restrictions in immune cell localization. Overall, our results reconcile with the hypothesis that the segment of the cranial nerve that is exposed to CSF flow is more susceptible to CCR2^+^ immune cell infiltration.

**Supplementary Information:**

The online version contains supplementary material available at 10.1007/s00401-025-02896-1.

## Introduction

Optic neuritis (ON) is a common feature in multiple sclerosis (MS) and other autoimmune demyelinating disorders such as neuromyelitis optic spectrum disorder (NMOSD) and myelin oligodendrocyte glycoprotein antibody-associated disease (MOGAD). ON is the most common initial clinical manifestation in ~ 30–60% adult patients of MOGAD and in ~ 20% MS patients [34, 38]. Symptoms such as hearing loss, trigeminal neuralgia, and olfactory dysfunction which involve the cochlear, trigeminal, and olfactory nerves, respectively, have also been reported in patients with these autoimmune demyelinating disorders, albeit at less frequency than optic neuritis [14, 18, 44, 59]. Another striking difference between the optic nerve and other cranial nerves in these autoimmune demyelinating disorders is the location of lesions. While optic neuritis has been commonly reported to be longitudinally extensive in MOGAD patients [1, 11], or as focal lesions mainly detected in the intra-orbital segment of the optic nerve in MS patients [34, 62]; the MRI lesion sites for trigeminal, facial, oculomotor, and cochlear nerves have been most frequently reported to be restricted to the root entry zone (REZ) of MS [20, 25, 36, 52], NMOSD [74], or MOGAD patients [13, 70].

Anatomically, there are two major differences between the optic nerve and other cranial nerves. Firstly, the optic nerve head is unmyelinated, while the rest of the nerve trunk is myelinated by oligodendrocytes [51]; therefore, the optic nerve is considered as an extension of the CNS. In contrast, other cranial nerves (except for the unmyelinated olfactory nerve) have been shown to have a central nervous system (CNS) segment and a peripheral nervous system (PNS) segment [15, 32, 71]. Secondly, the optic nerves are known to be ensheathed within three meningeal layers as they exit the skull, similar to the CNS where the subarachnoid space (SAS) filled with CSF is lined by the arachnoid mater and pia mater [45, 51]. However, the meningeal layers and SAS around other cranial nerves have not been closely examined; therefore, to what extent they are accessible to CSF flow is unknown.

Multiple lines of evidence from previous studies have suggested that CSF plays a role in determining inflammatory lesion sites in the CNS. In human studies, one study reported [3] that all NMOSD patients that exhibited leptomeningeal enhancement by MRI imaging also showed CSF pleocytosis, suggesting the potential role of CSF lymphocytes in the disease pathogenesis. CNS antigen (e.g., MOG, PLP, MBP) reactive T cells have also been detected in the CSF of MS patients with ON [72]. Multiple studies using experimental autoimmune encephalomyelitis (EAE) animal models have shown that the presence of meningeal T-cell infiltrates preceded the infiltration of T cells into the brain, spinal cord, and optic nerve [23, 79] and have proposed that the SAS is a major site by which effector cells invade the CNS parenchyma. In our previous study, we performed in vivo imaging in the MOG_35-55_-induced active EAE model and demonstrated that the typical spinal CSF flow in the cranial to caudal direction was impaired prior to the development of ascending immune cell infiltration that originated from the SAS of the sacral spinal cord. These findings indicated a role of CSF flow in determining the inflammatory lesion sites at the spinal cord [83].

Taken together, we hypothesize that accessibility to CSF predisposes cranial nerves to immune cell infiltration from the leptomeninges. We first report two unique clinical cases of inflammatory demyelinating disease, a patient with undefined type and a MOGAD patient, both of whom present with extensive optic neuritis and trigeminal nerve lesions at the REZ, with trigeminal nerve lesions appearing to terminate at the TZ. Consequently, we utilize the MOG_35-55_-induced active EAE model in *CX3CR1*^*GFP*^*/CCR2*^*RFP*^ reporter mice, to characterize the cranial nerve lesions and evaluate if CCR2^+^ immune cell infiltrates recapitulate the clinical observations in these two patients. Subsequently, by characterizing the anatomical arrangements of CNS barriers such as astrocyte foot processes and the arachnoid barrier along the nerves, along with functional analysis of the distribution of intracerebroventrically infused (i.c.v) CSF tracer, we aim to investigate the potential role of CSF in the localization of inflammatory sites at cranial nerves during neuroinflammation. Finally, we also assess the presence of lymphatic vessels around the investigated cranial nerves, which may implicate lymphatic clearance of immune cells in the resolution of inflammatory lesions.

## Material and methods

### MRI imaging of human patients with inflammatory demyelinating disease

MRI scans were performed using 3.0 T clinical scanners. The imaging details used in case 1 were as follows: images were acquired on a Skyra scanner (3.0 T, Siemens), while images for case 2 were obtained using a MAGNETOM Prisma scanner (3.0 T, Siemens). The imaging sequences included T2-weighted fat-saturated Dixon, 3D-T2-FLAIR, and Double Inversion Recovery (DIR). The imaging parameters for each sequence were as follows: T2-weighted fat-saturated Dixon: repetition time (TR) = 4500 ms, echo time (TE) = 116 ms, matrix size = 325 × 446, field of view (FOV) = 180 × 180 mm, and slice thickness = 3 mm. 3D-T2-FLAIR: TR = 7000 ms, TE = 392 ms, inversion time (TI) = 1800 ms, matrix size = 256 × 256, FOV = 240 × 240 mm, and slice thickness = 1 mm. Double Inversion Recovery (DIR): TR = 7500 ms, TE = 321 ms, TI1 = 3000 ms, TI2 = 450 ms, matrix size = 192 × 192, FOV = 240 × 240 mm, and slice thickness = 1.5 mm.

### Animals

*CCR2*^*RFP*^* / CX3CR1*^*GFP*^ micewere a kind gift from Dr. Israel F. Charo (UCSF, USA). Mice were bred heterozygous in house and kept in individually ventilated cages under specific pathogen-free conditions at a light–dark cycle of 13 h–11 h. Food and water were accessible ad libitum. EAE experiments were performed at 2–3 months of age and mice of both sexes were randomly assigned to experimental groups. *Prox1-GFP* [12] naïve mice of 8–12 weeks of age were also used. Our study examined male and female animals, and similar findings are reported for both sexes.

### Active experimental autoimmune encephalomyelitis (EAE) induction and scoring

EAE was induced by injecting an emulsion of myelin oligodendrocyte glycoprotein peptide_35–55_ (MOG_35‐55_ peptide, GenScript), 200 µg per animal, in complete Freund’s adjuvant (CFA, prepared from Incomplete Freund’s Adjuvant, Santa Cruz Biotechnology, USA, supplemented with Mycobacterium Tuberculosis, Difco). In brief, 100 µl emulsion of MOG_35-55_ and CFA was injected subcutaneously in mouse flanks (80 µl) and at the tail base (20 µl) at day 0 under short isoflurane anesthesia. Moreover, 400 ng PTX (List Biological Laboratories, Campbell, CA, USA) was applied intraperitoneally at day 0 and day 2 [78]. Wet chow was provided in a Petri dish as soon as the first mouse reached clinical onset. Two time points during EAE were investigated: EAE peak (clinical score 2, animals showing strong hindlimb paraparesis or paraplegia) and chronic (clinical score 1.5 ± 0.4, animals presenting partial hindlimb paraparesis after prior paraplegia, 17–24 days after EAE onset). Naïve mice were not subject to immunization procedures.

### Liposome infusion into the lateral ventricle

Mice were anesthetized with an initial intraperitoneal injection of ketamine (80 mg/kg) and medetomidine (0.4 mg/kg). After induction of deep anesthesia was confirmed by paw withdrawal reflex, for intracerebroventricular infusion (i.c.v), mice were given an additional injection of ketamine/medetomidine of 1/3 of the initial dose and fixed in a stereotactic frame (RWD, Mainz, Germany). The skull was thinned with a dental drill (F.S.T., North Vancouver, B.C., Canada) 0.92 mm lateral and 0.22 mm caudal from bregma. A 34G steel needle (Hamilton, Bonaduz, Switzerland) on a 10 µl gas tight syringe (Hamilton, Bonaduz, Switzerland) was inserted into the left lateral ventricle 2.35 mm ventral to the skull surface. DiD-labeled liposomes [81, 83] were formulated in house. Infusion of 3 µl liposomes at a rate of 0.5 µl/min was performed with a syringe pump (Stoelting, Wood Dale, IL, USA). The needle was left in place for 2 min before being retracted slowly to avoid backflow. The skull was closed with bone wax (Ethicon, Somerville, NJ) and the skin wound was closed with tissue glue (Vetbond™, Fisher Scientific, Reinach, Switzerland).

### Tissue processing

Naïve and EAE mice were subjected to transcardial perfusion with 10 ml PBS (Gibco, Paisley, UK) followed by 10 ml of 4% paraformaldehyde (PFA, Merk Darmstadt, Germany) in PBS. For each mouse, the cranium was harvested and post-fixed in 4% PFA for 24 h, then decalcified in 14% ethylenediaminetetraacetic acid (EDTA, Sigma-Aldrich, St. Louis, MO, USA) in PBS for 7–10 days. All tissues were immersed in 30% sucrose (Merk, Darmstadt, Germany) in PBS for 3 days for cryoprotection before being frozen at − 80 °C in O.C.T. (Tissue‐Tek®, Sakura Finetek, Umkirch, Germany). 30-µm thick sections were cut using a cryostat (CryoStar, NX50, Epredia, Cham, Switzerland) and were stored at -20 °C for later immunofluorescence staining.

### Immunofluorescence staining and confocal imaging

Frozen tissue sections were first hydrated with PBS for 10 min, then permeabilized by 0.1% triton for 10 min. 10% goat or donkey serum (depending on the species where secondary antibodies were raised) was used for blocking for 1 h at room temperature. Sections were incubated with primary antibodies with appropriate dilutions for 3 h at room temperature and then washed with PBS before incubating with appropriate secondary antibodies for 2 h at room temperature. Primary antibodies used in this study were as follows: glial fibrillary acidic protein (GFAP) (Rabbit polyclonal IgG, 1:200 dilution, Dako) and E-cadherin (Goat polyclonal IgG, 1:100 dilution, R&D systems). Secondary antibodies used in this study were as follows: Donkey anti-rabbit IgG Cy5 (1:300 dilution, Jackson ImmunoResearch Laboratories, Inc.) and Donkey anti-goat IgG Alexo Fluor 647 (1:500 dilution, Jackson ImmunoResearch Laboratories, Inc.). Overview of brain sections was imaged with a Zeiss AxioZoom V16 microscope. Higher magnification images were acquired with a  Zeiss LSM800 confocal microscope.

### Quantification of CCR2^+^ immune cell infiltrates

Decalcified cranial sections were randomly selected from three animals per experimental group and analyzed in three regions: the trigeminal nerve (7–10 sagittal sections), the cochlear nerve (8–13 sagittal sections), and the nerve fiber layer (NFL) above the cribriform plate (14–17 coronal sections). GFAP staining was performed on sagittal sections using the same reagents, and all images (1024 × 1024 pixel) were captured with a AxioZoom microscope under identical settings. Image analysis was conducted using Fiji software. For the trigeminal nerve, a circular region of interest (ROI) with a diameter of 200 pixels was placed at the CNS–PNS transition zone, centering on the apex of the curvature of the GFAP signal. A delineation was manually drawn using GFAP signal to distinguish CNS and PNS regions within the circle, with the user blinded to CCR2 signal. The raw integrated density (RawIntDen) of the CCR2 signal was measured in both CNS and PNS regions and normalized to tissue area (RawIntDen/area). A similar approach was used for the cochlear nerve, with an ROI defined by a circle of 150 pixels in diameter. For the nasal area, a 150 × 150 pixels square ROI was positioned above the cribriform plate within the tissue space between the olfactory bulbs, based on DAPI signal. The same quantification and statistical methods used for cranial nerves were applied.

### Statistical analyses

Statistical analyses were performed using GraphPad Prism 10 (GraphPad Software, LLC). Graphs and numerical values in the text represent mean ± SD. Normality and log-normality tests were performed, followed by log transformation of all data points to ensure a Gaussian distribution. Outliers were identified using the ROUT method and removed from further analysis. Means between two groups were compared using a two-tailed unpaired *t*-test, with *p* < 0.05 considered statistically significant.

## Results

### Case studies – two inflammatory demyelinating disease patients, both of whom presented with optic neuritis and trigeminal sensory impairment

The REZ in the literature is sometimes used as a synonym for CNS–PNS transitional zone (TZ), whereas in other publications the REZ is used to define the area where cranial nerves are attached to the brainstem with TZ included in this area [15, 26, 58, 84]. Here in this study, to avoid confusion, REZ refers to the later definition and is used to describe the MRI imaging findings. We used the term TZ to refer to the border between the CNS segment, where the nerve fibers are myelinated by oligodendrocytes, and the PNS segment, where the nerve fibers are myelinated by Schwann cells.

#### Case 1: an atypical inflammatory demyelinating case

**Fig. 1:**
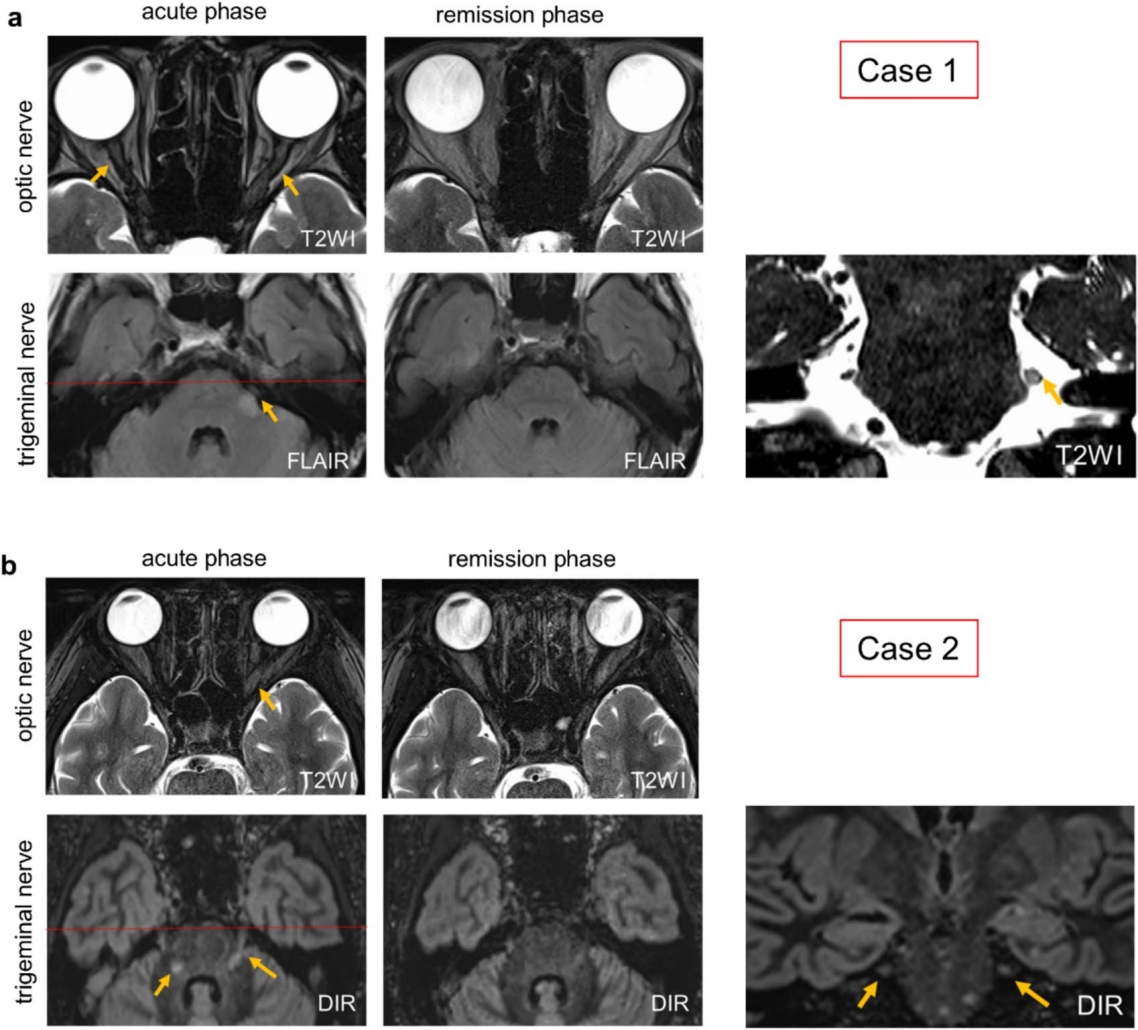
Two patients demonstrating both optic neuritis and trigeminal nerve lesions. **a** Axial T2 weighted images (T2W1) of the optic nerves showed bilateral hyperintensities (yellow arrows) at acute phase, which completely resolved at the remission phase in case 1 (a patient with inflammatory demyelinating disease of undefined type). Fluid attenuated inversion recovery (FLAIR) sequences showed a high signal intensity at the REZ of the left trigeminal nerve of this patient (yellow arrow) at acute phase, which partially resolved at remission phase. **b** Axial T2WI images showed a hyperintensity lesion of the left optic nerve at acute phase of case 2 (a MOGAD patient), which was nearly completely resolved at remission phase. The double inversion recovery (DIR) sequences showed bilateral lesions at the pons and the REZ of the trigeminal nerves that extended from the pons distally up to 4 mm. The trigeminal nerve lesions were partially resolved at remission phase. Coronal views (right panels) show the red scanned line corresponding to the axial view at the acute phase

A 70-year-old female developed hypoesthesia and an abnormal sensation in the left upper lip and cheek, which gradually extended over a day to involve the left forehead and periorbital region. Six days later, she experienced vision loss in her left eye over half a day, and on the ninth day, she woke up to complete blindness in her right eye. Brain MRI revealed bilateral optic nerve swelling with contrast enhancement and a lesion in the left intramedullary trigeminal tract at REZ with contrast enhancement (Fig. 1a, supplemental Fig. 1a, b). In addition, a periventricular lesion without contrast enhancement is observed (supplemental Fig. 1c). Both serum and cerebrospinal fluid were negative for aquaporin-4 (AQP4) antibodies (by ELISA and cell-based assay), and MOG antibodies were also negative (by cell-based assay). Cerebrospinal fluid analysis showed a cell count of 2/μL and elevated protein levels at 61.0 mg/dL. The IgG index was 0.59, and oligoclonal bands were negative. Myelin basic protein was markedly elevated, exceeding 2000 pg/mL. Due to the facts that this patient experienced only a single clinical episode and was of advanced age with an atypical periventricular lesion for MS, together with the above-mentioned laboratory findings, we consider this case as a neuroinflammatory demyelinating disease of undefined type. The symptoms improved with steroid pulse therapy, and imaging findings also showed partial resolution of the lesions. There were multiple discrete lesions on both optic nerves at the acute stage, extending from the cranial segment to the intra-orbital segment. The MRI lesion of the left trigeminal nerve at the acute stage extended from the root attachment point 3 mm distally before abruptly terminating. According to an early study [71], the TZ of human trigeminal nerve is located 3.0 mm away from the brainstem; therefore, we speculated that the trigeminal nerve lesion of this patient developed near the TZ.

#### Case 2: MOGAD patient

A 13-year-old female developed worsening headaches, eye pain, and vision loss over several days. One month later, she experienced right lower limb weakness, gait disturbance, and decreased sensation on the left side of her tongue. Brain MRI revealed a longitudinally extensive high-intensity lesion in the left optic nerve (longitudinal length = 39 mm) (Fig. 1b). Bilateral trigeminal nerve lesions extended peripherally 4 mm beyond the pons. Serum MOG antibodies were positive. Her acute symptoms worsened despite steroid pulse therapy but improved with plasma exchange. The MRI signal abnormalities were partially resolved. Recurrences were successfully suppressed with steroids and immunosuppressants. Based on the MRI lesions location, we speculated that the trigeminal nerve lesions of this patient formed around or slightly beyond the TZ.

### EAE mice present with a restricted CCR2^+^ cell infiltration at the TZ in trigeminal and cochlear nerves

To investigate if EAE mice present lesions at the TZ zone of cranial nerves, we induced active EAE with MOG_35-55_ peptide in double knock-in *CCR2*^*RFP*^* x CX3CR1*^*GFP*^ mice [64]. This double-reporter mouse allows us to differentiate CNS resident macrophages (microglia and border-associated macrophages) from blood-borne monocyte-derived cells during EAE [42, 83]. At the peak stage of EAE, mice were perfused, and each of the cranium was decalcified. Sagittal cryosections were used to assess the trigeminal and cochlear nerves, whereas coronal sections were utilized to investigate the optic and olfactory nerves (Supplemental Fig. 2 a, b).

**Fig. 2:**
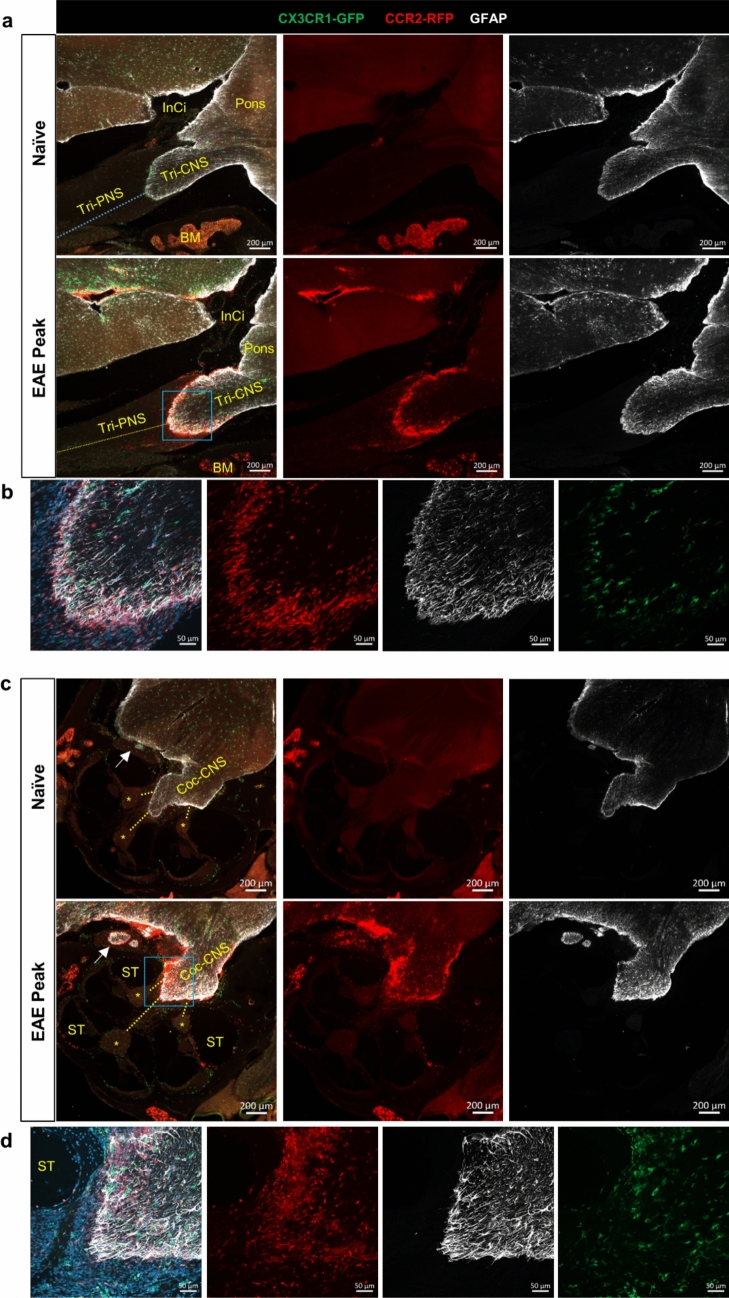
Confined CCR2 + cell infiltration at the TZ of trigeminal and cochlear nerves in EAE mice. **a** No CCR2^+^ cells were apparent in naïve mice, but CCR2^+^ cells were preferentially associated with GFAP^+^ astrocyte foot processes at the TZ of the trigeminal nerve in EAE mice. Images are representative of *n* = 3 mice in each group. Tri-PNS, peripheral segment of the trigeminal nerve; Tri-CNS, CNS segment of the trigeminal nerve; InCi, interpeduncular cistern; BM, bone marrow. **b** Confocal images of the boxed area in **a**, showing the association of CCR2^+^ cells with the TZ marked by the dome-shaped line of GFAP^+^ astrocyte foot processes and CX3CR1^+^ microglia. **c** CCR2^+^ cells were not seen at the cochlear nerve in naïve mice, but CCR2^+^ cells were preferentially associated with GFAP^+^ astrocyte foot processes at the TZ of the cochlear nerve in EAE mice. Images are representative of *n* = 3 mice in each group. Cross-sections of the facial nerve (white arrows) are visible, with strong association of CCR2^+^ cells with the GFAP staining in the EAE mouse. Coc-CNS, the CNS segment of the cochlear nerve; ST, scala tympani; *, spiral ganglion; dotted lines, peripheral segment of the cochlear nerve bundles. **d** Confocal images of the boxed area in **c**, showing the association of CCR2^+^ cells with the TZ. The CX3CR1^+^ microglia were diffusely distributed, unlike the dome-shaped distribution seen at the TZ of the trigeminal nerve. *ST*, scala tympani

The TZ transition zone has also been reported to be equipped with fibrillary astrocytes [71], and the line of glia cell transition takes the form of a dome. This dome-shaped line of astrocyte foot processes is sometimes referred to as the glia limitans of the cranial nerves [55]. GFAP immunofluorescence staining for the astrocyte foot processes has been previously shown to mark the central to peripheral TZ at cranial nerves [32, 80]. Using GFAP staining we confirmed the previously described dome-shaped TZ. Contrary to an early study where microglia were not observed in the nerve trunk of human cadavers [71], we observed a dome-shaped line of CX3CR1^+^ microglia overlapping with the GFAP staining at the TZ of the trigeminal nerve and diffuse distribution of CX3CR1^+^ microglia within the cochlear nerve (Supplemental Fig. 3). Interestingly, sagittal sections of EAE mice assessed for trigeminal and cochlear nerves showed that CCR2^+^ cells were not evenly distributed along the nerve trunk. Instead, CCR2^+^ cells had largely accumulated at the surface of the nerve trunk proximal to the REZ, but across the nerve parenchyma only at the TZ, also forming a dome-shaped distribution pattern (Fig. 2a-d). Confocal images showed that CCR2^+^ cells were distributed not only on the CNS side of the dome-shaped astrocyte border but also on the peripheral segment near this border (Fig. 2b, d). These results indicate that the cranial nerve inflammatory lesions in this active EAE model resemble those seen by MRI in patients with inflammatory demyelinating diseases.

In addition, we observed CCR2^+^ cell accumulation inside the SAS near cranial nerves. For example, near the trigeminal nerve, CCR2^+^ cells were clearly accumulated inside the interpeduncular cistern which is a basal subarachnoid cistern located in the interpeduncular fossa, anterior to the pons (Fig. 2a). In addition, the scala tympani of the cochlea is known to be a fluid compartment that is connected to the SAS through the cochlear aqueduct. CCR2^+^ cells were found on the wall of the scala tympani of all EAE mice (Fig. 2c), but not in the naïve mice, strongly suggesting that CSF flow mediates immune cell infiltration into the cochlea.

### EAE mice present with broad CCR2^+^ cell infiltration in optic and olfactory nerves

**Fig. 3:**
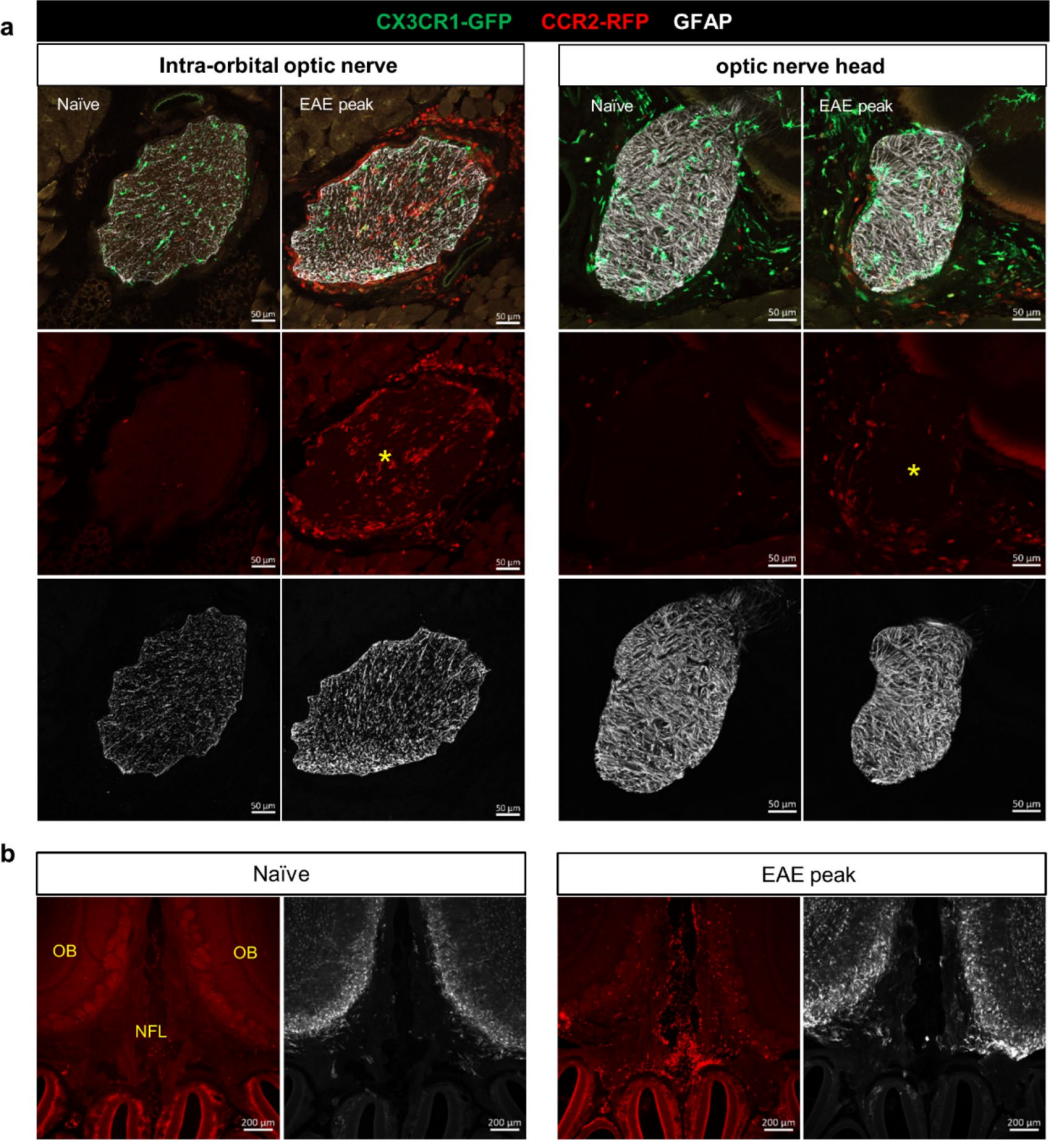
Widespread distribution of CCR2^+^ cells within the optic and olfactory nerves. **a** GFAP staining revealed two distinct patterns of astrocyte morphology between the intra-orbital portion and the optic nerve head. There was no clear difference between the naïve and the EAE mice in GFAP staining. Images are representative of *n* = 3 mice in each group. In EAE mice, CCR2^+^ cells were diffusively distributed within the intra-orbital segment, but were nearly devoid of the optic nerve head, where astrocytes form a dense network. *, optic nerve. **b** CCR2^+^ cells were abundantly accumulated between the two olfactory bulbs (OB), within the nerve fiber layer (NFL), where sparse GFAP labeling was seen. There were abundant GFAP^+^ astrocytes around the glomeruli of the olfactory bulb. Images are representative of *n* = 3 mice in each group

Next, we performed GFAP staining on decalcified coronal sections to assess the optic and olfactory nerves. Previous studies have identified two areas in the mouse optic nerve where astrocytes are differentially organized. In the region of optic nerve head where ganglion cell axons are unmyelinated, the GFAP-labeled processes are particularly dense and are transversely oriented with respect to the long axis of the nerve, forming the so-called glial tubes to partite axon bundles [75, 76]. In the myelinated portion of the optic nerve, astrocytes exhibit various shapes, sizes and orientation [8, 9]. Using GFAP immunofluorescence staining, we confirmed these previous findings by revealing that astrocyte foot processes form two morphologically distinct structures between the optic nerve head and the remaining portion of the nerve. In parallel, CCR2^+^ cells were nearly devoid at the optic nerve head, where dense tubular astrocytic processes exist, but the remaining optic nerve section was inhabited by abundant CCR2^+^ cells (Fig. 3a). This result indicates that the optic nerve head in this EAE model is protected from CCR2^+^ immune cell infiltrates, which may be mediated by a physical barrier formed by the astrocytes processes.

GFAP staining of the olfactory nerve bundles revealed sparse staining in the NFL, but strong staining around the glomeruli (Fig. 3b). In parallel, abundant CCR2^+^ were accumulated in the NFL, but not around the glomeruli. Pathways along the olfactory nerve bundles have been identified as a major CSF efflux route in the mouse [48, 61, 73, 85]. CCR2^+^ cell accumulation in this CSF-filled compartment is further evidence that the cranial nerve segment that is exposed to CSF flow is more susceptible to immune cell infiltration. It is likely that the lack of a glia barrier permits immune cell infiltration into this region.

Taken together, GFAP staining of the investigated cranial nerves supports the hypothesis that CSF may mediate immune cell distribution to the cranial nerves, and that astrocytes may provide a physical barrier to prevent immune cells infiltration into the cranial nerve parenchyma from the CSF space, as well as limiting their spread across the TZ of the nerve.

### CCR2^+^ cells are highly accumulated at the segment of cranial nerves that is accessible to CSF flow

To further validate our hypothesis that CSF plays a role in disseminating CCR2^+^ immune cells into cranial nerves, we used 106-nm DiD-labeled liposomes as a CSF tracer, which has been shown to be optimal in assessing the CSF flow, as they do not enter the CNS parenchyma unless the glia limitans superficialis is severely damaged [83].

**Fig. 4:**
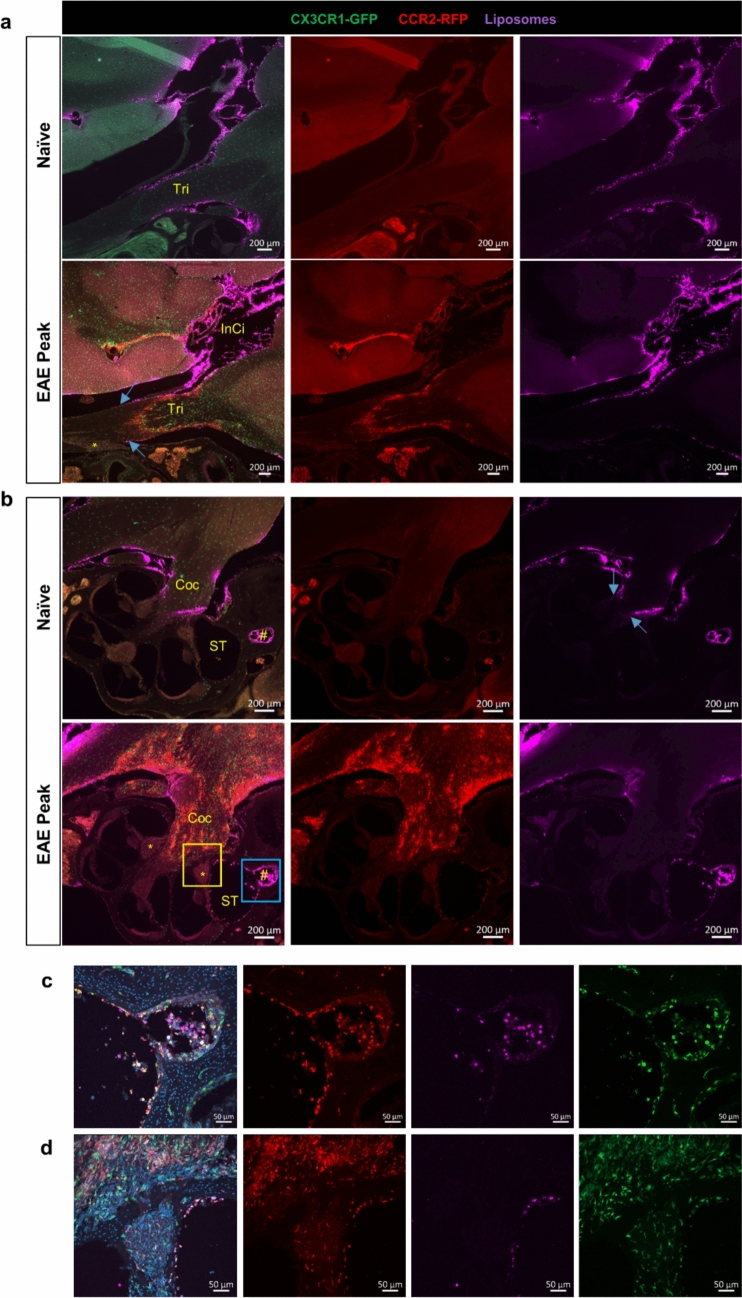
CCR2^+^ cells largely accumulated at the CSF accessible segment of trigeminal and cochlear nerves during EAE. **a** Representative images (*n* = 3 mice in each group) show that liposomes distributed along the trigeminal nerve only slightly beyond the TZ toward the PNS in both naïve and EAE mice. CCR2^+^ cells were not visible at the PNS segment, where there is also no liposome distribution on the nerve surface. Tri, trigeminal nerve; InCi, interpeduncular cistern. **b** Representative images (*n* = 3 mice in each group) show that the liposome accessible segment of the cochlear nerve (Coc) presented with substantial infiltration of CCR2^+^ cells. Liposomes were visible in the cochlear aqueduct in the naïve and EAE mice, but CCR2^+^ cells were only found at this location in EAE mice. The basal scala tympani (ST) is directly connected with the cochlear aqueduct (#). CCR2^+^ cells were not seen in the spiral ganglia of the naïve mice, but were visible in some of the ganglia (*) in the EAE mice. **c** Confocal images of the blue box area in **b**. **d** Confocal images of the yellow box area in **b**

For the trigeminal nerve, 2 h post-i.c.v infusion, liposomes were found distributed along the CNS segment of the nerve trunk as far as to the peripheral segment where CCR2^+^ cells also reached their furthest point along the nerve trunk (Fig. 4a). Unlike CCR2^+^ cells that were also inside the nerve parenchyma, liposomes were not observed inside the nerve parenchyma and instead remained on the surface of the nerve. These observations indicate that while CSF flow may facilitate propagation of CCR2^+^ cells along the SAS of the cranial nerves, other mechanisms (e.g., chemokine-mediated cell infiltration) are likely involved in the entrance of CCR2^+^ cells into the nerve parenchyma [35]. Given that liposomes can access the peripheral nerve further than the dome-shaped glia limitans, we reason that this short section of the peripheral segment of trigeminal nerve may be a weak point for CCR2^+^ cell infiltration.

Regarding the cochlear nerve (Fig. 4b-d), liposomes seem to be largely accumulated at the entrance of the internal acoustic canal from the cochlea, where CCR2^+^ cells also had accumulated. CCR2/CX3CR1 double-positive cells were apparent in the spiral ganglia of EAE mice, but not in healthy mice. Nevertheless, as expected, liposomes spread through the cochlear aqueduct into the scala tympani. In EAE mice, numerous CCR2^+^ cells, positive for liposomes, were found inside the cochlear aqueduct and on the wall inside the scala tympani. These observations support the hypothesis that CSF flow may contribute to the spreading of immune cells into the inner ear through the cochlear aqueduct.

**Fig. 5:**
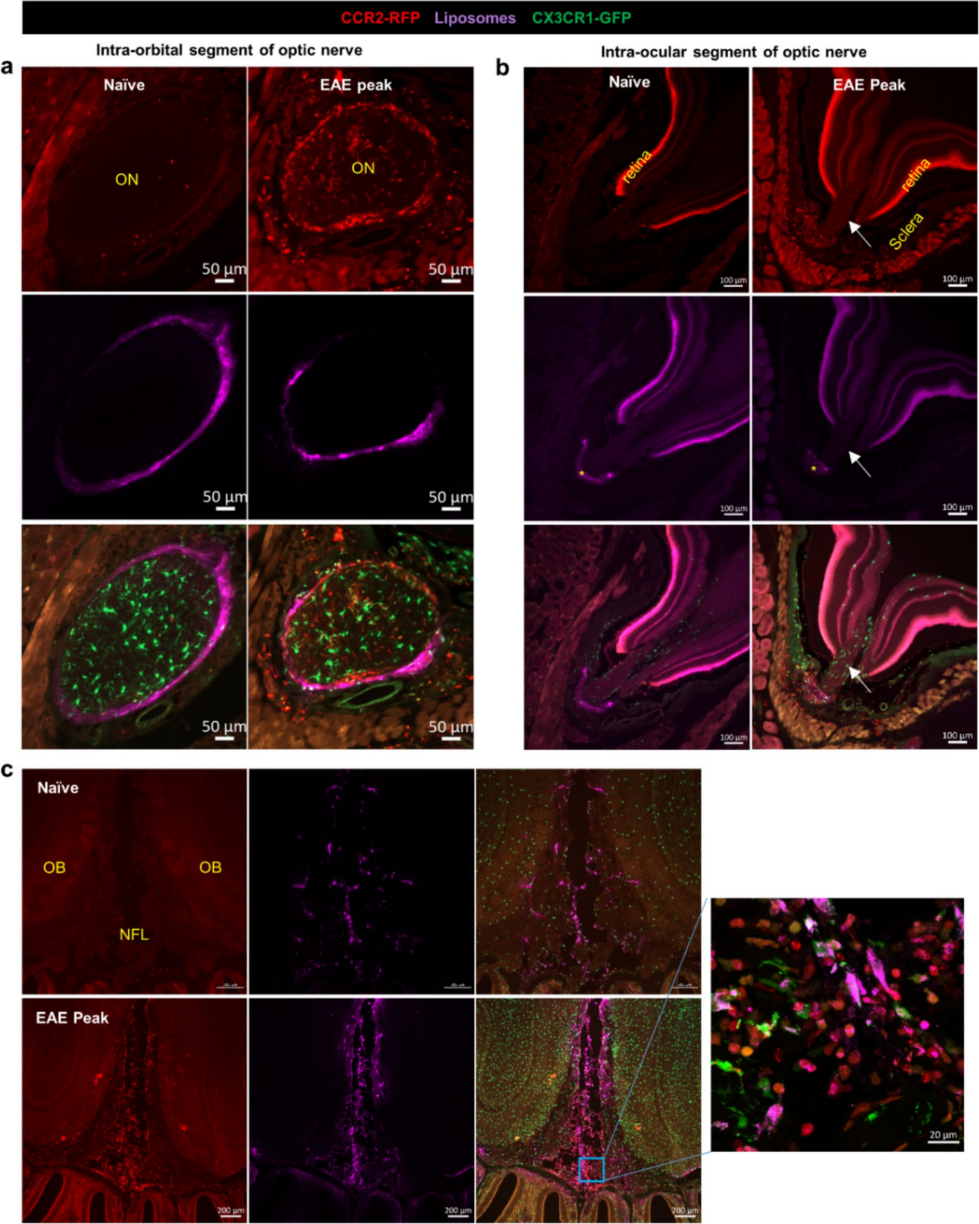
The CSF accessible portion of the optic and olfactory nerves are heavily infiltrated with the CCR2^+^ cells during EAE. **a-b** Representative images (*n* = 3 mice in each group) showing widespread distribution of CCR2^+^ cells in the intra-orbital segment of the optic nerve, in contrast to the lack of CCR2^+^ infiltrates in the intra-ocular segments (white arrow) of the optic nerve. Liposomes were around the optic nerve at the intra-orbital segment, accumulated around the sclera (*), but no liposomes were seen around the intra-ocular segment of the optic nerve (white arrow). **c** Representative images (*n* = 3 mice in each group) of the colocalization of CCR2^+^ cells with liposomes within the NFL between the two olfactory bulbs (OB) during EAE. A confocal image from the blue box area is shown on the right

Optic nerves have been shown to be encased in the SAS throughout their length up until the sclera in mice [45]. As expected, the liposomes spread along the optic nerve up to this point (Fig. 5a, b), where they form a cuff-like distribution. CCR2^+^ cells were not found in the intra-ocular segment, where liposomes were also absent. However, numerous CCR2^+^ cells were found inside the parenchyma of the remaining portion of the optic nerve, where liposomes were distributed along the nerve surface.

Olfactory nerves have been shown by multiple studies to be enclosed by a discontinuous arachnoid barrier layer, providing a potential mechanism for CSF to “leak” into the NFL [31, 73]. There was substantial colocalization of CCR2^+^ and liposomes at the NFL of the olfactory nerves (Fig. 5c), but once the olfactory nerve bundles pass through the cribriform plate, CCR2^+^ were only occasionally found around the nerve bundles in the nasal submucosa.

To further support our hypothesis that the CCR2^+^ cells infiltrated into the cranial nerves are largely originated from the SAS, we checked multiple SAS cisterns in the EAE mice, found that abundant CCR2^+^ cells colocalized with liposomes (Supplemental Fig. 4). In summary, the i.c.v-infused liposomes are significantly colocalized with CCR2^+^ cells within the SAS.  During EAE, the cranial nerve segments that are accessible to liposomes are also inhabited with abundant CCR2^+^ infiltrates.

### Differential arachnoid coverage among cranial nerves

Previous studies of the leptomeninges have shown that the outer cell layer of the arachnoid (also known as the arachnoid barrier layer) expresses the adherens junction protein E-cadherin; therefore, this marker is used to indicate the outer border of the SAS [16, 50, 60]. The proposal of a so-called Prox1^+^ SLYM layer has evoked discussion and disagreement among researchers [50, 53, 60]. Some researchers referred to this Prox1^+^ layer as a fourth layer of meninges forming a barrier between an inner and outer SAS, while others provided evidence that this is the inner arachnoid layer directly adjacent to the E-cadherin^+^ barrier layer. Consequently, we performed E-cadherin staining on decalcified cranial tissue sections of the Prox1-GFP reporter mice that were i.c.v infused with liposomes. We speculated that liposomes would stop spreading along the nerve trunk at the point where the arachnoid barrier layer terminates on the nerve.

**Fig. 6:**
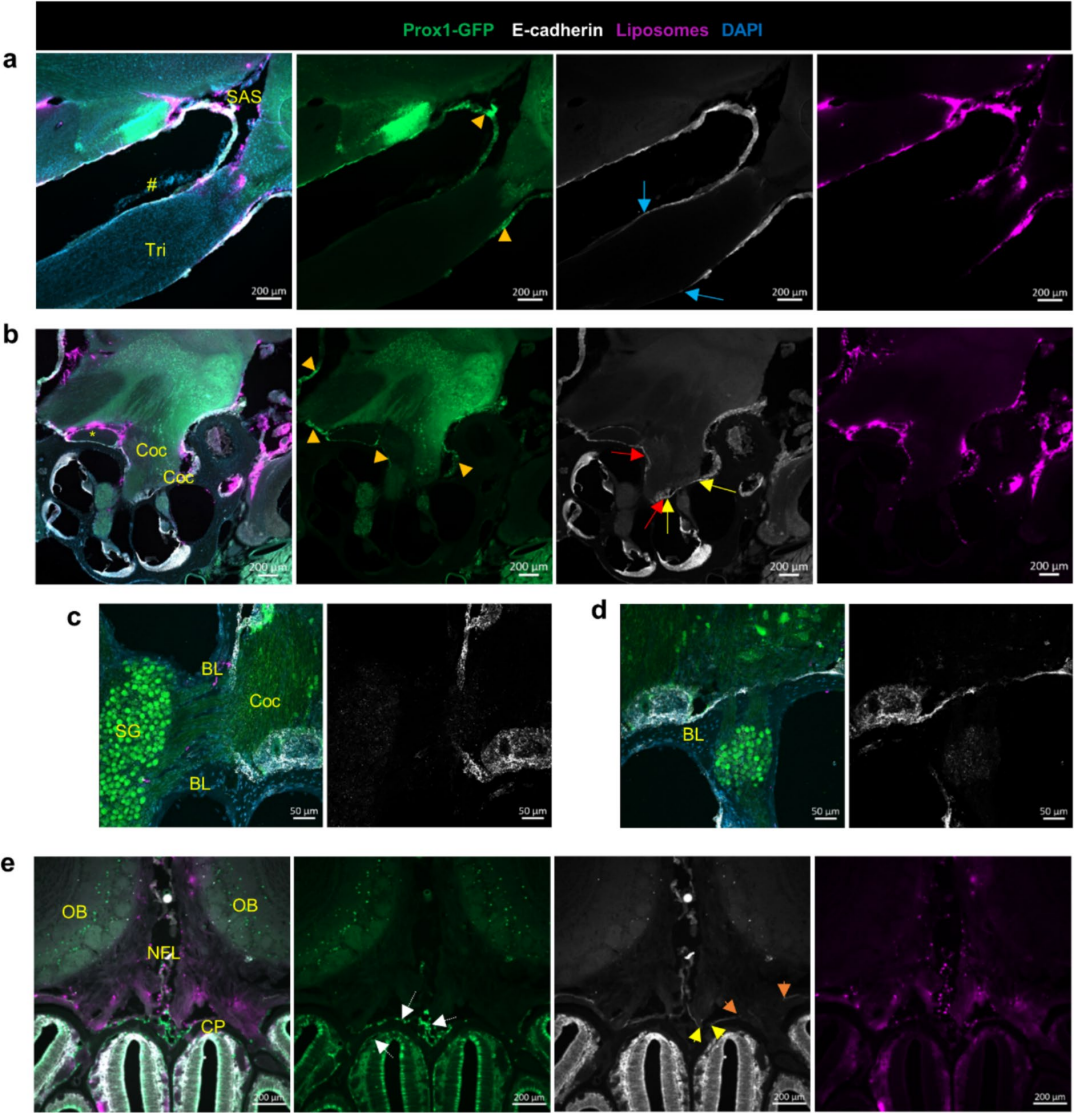
Trigeminal and cochlear nerves are only partially accessible to CSF. **a** Representative images showing that the Prox1^+^ inner arachnoid layer (arrowhead) is closely associated with the E-cadherin^+^ arachnoid barrier layer, which lines the basal brain and forms a SAS around the trigeminal nerve (Tri). Both arachnoid layers end at the point (blue arrows) where the distribution of CSF-injected liposomes also terminate. #, dura mater. **b** Representative images showing that the Prox1^+^ inner arachnoid layer (arrowhead) is closely associated with the E-cadherin^+^ arachnoid barrier layer around the facial nerve (*) and the cochlear nerve (Coc) to form a SAS that liposomes could also access. **c** Confocal images of the area between the two red arrows shown in **b**. The Prox1^+^ and E-cadherin^+^ layers are interrupted at the point where the cochlear nerve bundles pass through the bony labyrinth (BL) to reach the spiral ganglia (SG). Liposomes were visible between the nerve bundles. **d** Confocal images of the area between the two yellow arrows shown in **b**. Prox1^+^ and E-cadherin^+^ layers are interrupted at the point where  two cochlear nerve bundles merge into the trunk of the cochlear nerve. **e** Representative images showing discontinuous E-cadherin^+^ layer and the lack of the Prox1^+^ layer (between the yellow arrowheads and between the orange yellow arrowheads) where olfactory nerve bundles pass through the cribriform plate (CP). Liposomes were visible on both side of the CP. Prox1^+^ lymphatic vessels (white arrows) closely associated with the CP are visible. Images are representative of *n* = 4 naïve mice

Interestingly, E-cadherin staining on the trigeminal nerve revealed an arachnoid cul-de-sac around the nerve before the ganglion (Supplemental Fig. 5a). No spreading of the liposomes was detected beyond this point toward the peripheral segment of the trigeminal nerve (Fig. 6a). The Prox1^+^ layer was immediately adjacent to the E-cadherin layer at the basal surface of the brain. At the point where the trigeminal nerves emerge from the pons, both layers reflect off the brain surface to form the interpeduncular cistern. The Prox1^+^ and E-cadherin^+^ layers then together form the outer boundary of a sheath around the trigeminal nerve that ends in the cul-de-sac. This observation indicates that similar to  the surface of the brain, Prox1 marks the inner arachnoid layer, instead of a lymphatic-like fourth layer of meninges around the trigeminal nerve as suggested [37]

Similarly, there was also a Prox1^+^ layer directly adjacent to the E-cadherin^+^ layer around the cochlear nerve (Fig. 6b). Surprisingly, where the cochlear nerve bundles originating from the spiral ganglion pass through the bony labyrinth of the cochlear to merge into the cochlear nerve trunk, the E-cadherin^+^ layer is discontinuous (Fig. 6c, d), which microscopically looks similar to the olfactory nerve bundles when they pass through the cribriform plate (Fig. 6e). In some tissue sections, we indeed observed that liposomes spread into the cochlear nerve bundles and spiral ganglion, indicating that the CSF from the SAS of the cochlear nerve can enter the cochlea (Fig. 6c, d). Nevertheless, different from the olfactory nerve bundles, which are surrounded by a rich network of lymphatic vessels both above and below the cribriform plate (Fig. 6e), we did not observe Prox1^+^ lymphatic vessels near the cochlear nerve or trigeminal nerve.

### E-cadherin^+^ arachnoid layer remains morphologically unchanged during EAE

Next, we compared E-cadherin staining around cranial nerves in naïve and EAE mice (Supplemental Figs. 6 and 7), to check if the arachnoid barrier is disrupted during EAE to allow immune cells to spread to the surrounding tissue. For all the investigated cranial nerves (trigeminal, cochlear, optic, and olfactory nerves), we did not observe any morphological differences between the naïve and EAE group based on the staining of E-cadherin. Consistent with the above-mentioned CSF tracer study, the CCR2^+^ cells were  found in the spiral ganglia and below the cribriform plate likely due to the discontinuity of the arachnoid barrier layer. Another note of interest is that abundant CCR2^+^ cells were  found within the periorbital fat tissue beyond the E-cadherin^+^ arachnoid barrier layer, even though the E-cadherin staining did not reveal any differences between the naïve and EAE mice (Supplemental Fig. 7a). This is consistent with clinical MRI observations [11, 68] that MOGAD patients often present with characteristic lesions in the peribulbar fat (also referred to as perineural enhancement). At this stage, we cannot rule out the possibility that the arachnoid barrier layer does not form a barrier at the optic nerve head, which may allow immune cells within the nerve sheath to access the surrounding tissue.

### CCR2^+^ cells at the TZ were significantly reduced at chronic stage

**Fig. 7:**
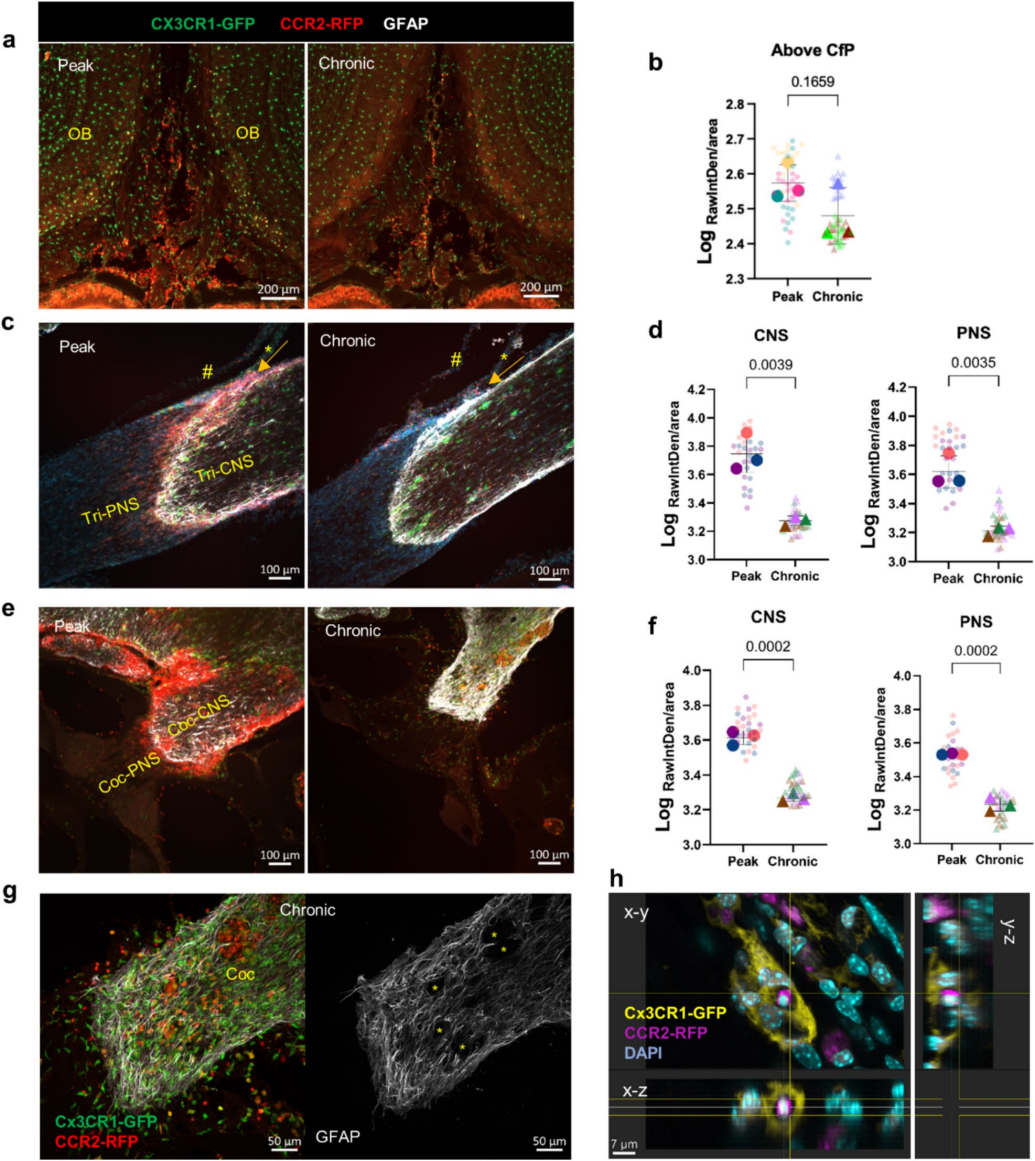
Decreased CCR2^+^ cells around the trigeminal and cochlear nerves during the chronic stage of EAE. **a, b** Representative images and quantification showing the levels of CCR2^+^ infiltratesabove the cribriform plate (Cfp) at EAE peak and chronic stage. **c, d** Representative images and quantification comparing CCR2^+^ cells on the CNS side (Tri-CNS) and the PNS side (Tri-PNS) of the trigeminal nerve at EAE peak and chronic stage. It should be noted that at EAE chronic stage, numerous CCR2^+^ cells had accumulated within the subarachnoid space (arrow). #, dura mater; *, arachnoid mater. **e, f** Images and quantification comparing CCR2^+^ cells on the CNS side (Coc-CNS) and the PNS side (Coc-PNS) of the cochlear nerve at EAE peak and chronic stage. **g** Confocal images showing that at the EAE chronic stage, CCR2^+^ cells accumulations were present in GFAP-negative cavities. **h** A representative confocal image showing the engulfment of CCR2^+^ cell by CX3CR1^+^ microglia. Quantifications are performed from *n* = 3 mice in each group, 14–17 tissue sections/mouse

Previous studies have suggested that the lymphatic vessels near the cribriform plate are important for immune cell migration out of the CNS during neuroinflammation [31]. We  found Iba1^+^ macrophages inside Prox1^+^   cribriform plate lymphatic vessels in naïve mice, while CCR2^+^ cells were found inside LYVE1^+^ lymphatic vessels in EAE mice (Supplemental Fig. 8). At chronic EAE, the CCR2^+^ cells appeared to have reduced numbers above the cribriform plate; however, this decrease did not reach a significant difference from the levels seen at peak disease (Fig. 7 a, b). Both trigeminal and cochlear nerves showed a significant reduction of CCR2^+^ cells on the CNS and PNS side at chronic EAE (Fig. 7c–f). Since we did not find lymphatic vessels near the TZ of cochlea and trigeminal nerves, lymphatic vessel-mediated migration of immune cells along these nerves out of the CNS is currently debatable.

Furthermore, with the significantly reduced number of CCR2^+^ infiltrates, confocal imaging showed “black holes” within the fibrillary branches of astrocytes. Interestingly, CCR2^+^ cells were found in these holes in the cochlear nerves of 3 out 3 EAE mice (Fig. 7g). This phenomenon was only observed in one of the 3 mice for the trigeminal nerves. Activated microglia have been previously shown to phagocytose dying and apoptotic cells neurons and oligodendrocytes [27, 56, 57]. However, it is still unclear whether CX3CR1^+^ microglia are capable of phagocytosing CCR2^+^ cells during neuroinflammation. We indeed observed microglia engulfment of CCR2^+^ cells inside the cochlear nerves (Fig. 7h).

Based on these results, while we cannot rule out a role for cribriform plate lymphatics in the drainage of CCR2^+^ cells from the SAS, the mechanism for CCR2^+^ immune cell resolution at the EAE chronic stage for trigeminal and cochlear nerves remains to be elucidated.

### Graphical summary

**Fig. 8:**
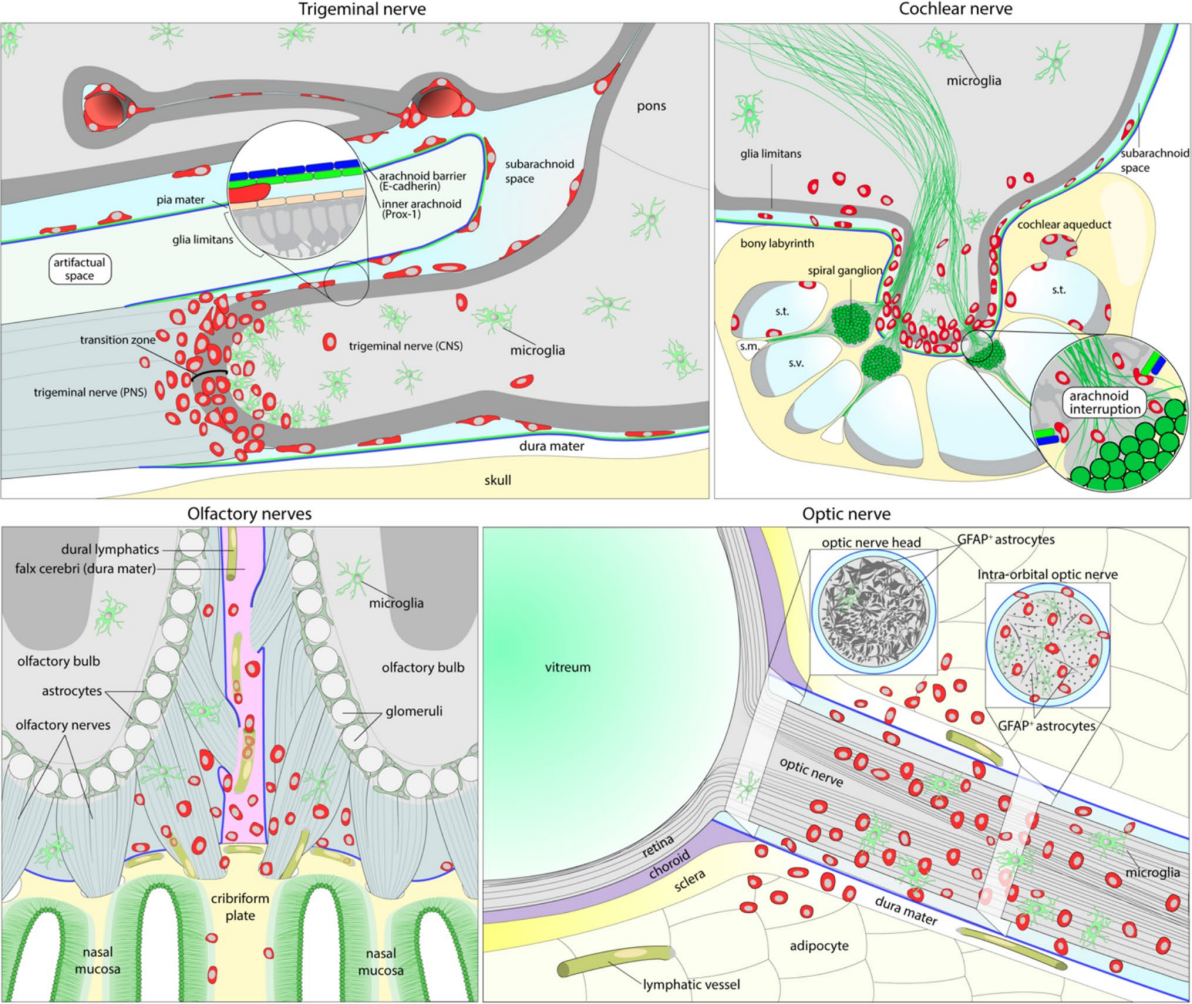
Graphical summary of the findings at EAE peak stage. CCR2^+^ cells (red-colored cells) infiltration at the trigeminal, cochlear, olfactory, and optic nerves and their association with relevant anatomical structures illustrated on the schematic. *s.t*. scala tympani, *s.m*. scala media, *s.v*. scala vestibuli

The key histopathological findings of the trigeminal, cochlear, olfactory, and trigeminal nerves from the EAE mice at peak disease are summarized schematically (Fig. 8), highlighting the CCR2^+^ infiltrates, SAS, the differential arrangement of inner and external arachnoid matter, glia limitans marked by GFAP^+^ astrocyte foot processes for trigeminal and cochlear nerves, as well as specially arranged/distributed GFAP^+^ astrocytes in optic nerve and olfactory nerves and the distribution of CX3CR1^+^ microglia.

## Discussion

In this study, we report two clinical cases confirming that trigeminal nerve lesions can be localized at the REZ in patients with inflammatory demyelinating disease. To explore the mechanisms underlying this phenomenon, we used a MOG_35-55_-induced active EAE mouse model in *CX3CR1*^*GFP*^*/CCR2*^*RFP*^ reporter mice. We show that CCR2^+^ immune cell lesions develop particularly at the TZ of the trigeminal and cochlear nerves. We then describe how the differential anatomical features of the glia and arachnoid barriers of the trigeminal, cochlear, optic, and olfactory nerves may affect the localization of cranial nerve lesions. Applying a liposomal CSF tracer into the lateral ventricle in EAE mice, we demonstrate that CCR2^+^ cells were largely confined to the cranial nerve segment that is accessible to CSF. At the chronic stage of EAE, CCR2^+^ immune cell infiltrates were largely resolved from the TZ of trigeminal and cochlear nerves through mechanisms that deserve further exploration. In summary, the current study indicates that the MOG_35-55_-induced active EAE model provides an invaluable tool to study the underlying mechanisms of cranial nerve lesions in autoimmune neuroinflammatory diseases. Furthermore, the involvement of CSF in determining the seeding of lesions in cranial nerves provides the groundwork for future exploration of CSF-mediated delivery of therapeutic interventions.

MS is an autoimmune-mediated neuroinflammatory demyelinating disease. In contrast to optic neuritis secondary to MS, otolaryngologic symptoms associated with MS, such as trigeminal neuralgia, hearing loss, tinnitus, and smell alteration, have traditionally been considered to be much less common. However, multiple review articles in recent years have noted that trigeminal neuralgia and auditory symptoms are more common than originally thought and may often be  overlooked [17, 19, 20, 40]. MOGAD is a relatively new clinical entity with a predisposition to the optic nerves and spinal cord, yet other cranial nerve involvement has also been reported in MOGAD patients with gadolinium enhancement often observed at the REZ/TZ during MRI examinations [13, 21, 39, 63].

While the initiating CNS antigen is still not clear in the case of MS,MOG and AQP4 have been considered to be the main targets of CNS destruction by autoantibodies during MOGAD and NMO, respectively. One reason that the optic nerves are more frequently and extensively involved in these demyelinating diseases may simply be because the optic nerves are part of the CNS; thus, autoreactive immune cells that have entered the CNS may freely access their target. However, some of the anatomical features of the optic nerves do not reconcile with this concept. For example, the optic nerve head (also known as the optic disc) often shows swelling in MOGAD as well as in NMO patients. This is despite the fact that the optic nerve head of humans and other species is unmyelinated (thus lacks MOG) and has been found with minimal expression of AQP4 [54]. Furthermore, spinal nerve root enhancement by gadolinium has also been reported in MRI imaging of MOGAD patients, despite the fact that MOG has not been found to be expressed at the human spinal nerve roots [24]. These unsettled discrepancies prompted us to consider whether other possible mechanism(s) may be involved in determining the locations of inflammatory lesions along nerves.

The MOG_35-55_-induced active EAE model has been largely considered as an animal model of MS by recapitulating some of the clinical features, such as BBB breakdown, immune cell infiltration, and optic neuritis. Accumulating evidence suggests that this EAE model also shares many similar pathological features to MOGAD, e.g., multiple pathophysiological features involving the visual system [10], leptomeningeal lesions, and frequent medullary cone involvement [4, 69, 83] despite the general acceptance that antibodies originating from mouse immunization with the MOG_35-55_ peptide are non-pathogenic [28, 46, 47]. Our previous study using this EAE mouse model documented leptomeningeal vessel leakage of fibrinogen, with immune cells first apparent in the spinal SAS before they reached the spinal cord parenchyma [83]. Furthermore, the sacral spinal cord (medullary cone) showed more severe immune cell infiltrates at an earlier stage of disease than the rostral spinal cord segments. The current study demonstrates that the site of inflammatory lesions in the trigeminal nerve in the MOG_35-55_-induced EAE model recapitulates that of patients with inflammatory demyelinating disease. This suggests that lesions in the trigeminal nerve, and perhaps also in other cranial nerves, are caused by non-antibody-mediated mechanism(s) that the MOG_35-55_ EAE model has in common with patients with inflammatory demyelinating diseases.

In the current study, CCR2^+^  immune cells were localized along all four cranial nerves investigated but were found at specific locations depending on the involved nerve. In contrast to the widespread distribution along the optic and olfactory nerves, CCR2^+^ cells characteristically accumulated around the TZ of the trigeminal and cochlear nerves. This suggested that the arrangement of the meninges along each nerve is different and likely plays a role in CSF-mediated seeding of the immune cells.

The arrangement of the meningeal sheaths around different cranial nerves is not well understood. A recent study using two different sizes of gold nanoparticles (1.9 mm and 15 mm) infused into the lateral ventricle demonstrated that gold nanoparticles were found on the PNS side of trigeminal nerve, and this phenomenon was size and time dependent [41]. The authors of this study concluded that CSF flows to peripheral nerves. The term “flow” in the fluid dynamics field is interchangeably used with bulk flow or convective flow, meaning a movement of fluid down a pressure gradient, independent of molecular size. Our results with the E-cadherin staining showed that the arachnoid barrier layer forms a cul-de-sac around the trigeminal nerve at the PNS segment near the TZ. The arachnoid cul-de-sac around the trigeminal nerve is similar to what have been described by Brierley et al. for spinal cord nerve roots [6, 7]. When India ink (estimated size of 0.4–1.5 µm) was infused into the lateral ventricle, the India ink particles accumulated around the spinal nerve roots to form ink cuffs. Similarly, we infused 106 nm liposomes into the lateral ventricle and 2 h later observed the accumulation of liposomes at the trigeminal arachnoid cul-de-sac, with no liposomes apparent inside the nerve parenchyma, indicating that CSF flow along the trigeminal nerve ends at the point. The termination of the arachnoid barrier layer (and thus the SAS) at the CNS segment of trigeminal nerve is consistent with the abundance of CCR2^+^ infiltration at this site. This prompted us to speculate that CCR2^+^ cells within the CSF are funneled into the arachnoid cul-de-sac, from where they are able to readily migrate into the nerve parenchyma and PNS of the trigeminal nerve due to the lack of a glia limitans at this location.

The inner ear allows direct fluid communication with the CSF through the cochlear aqueduct, which opens into the scala tympani of the cochlear apparatus. Another potential connection has been suggested to be through the perineural space of the cochlear nerve [49, 77]. The authors of an elegant systematic review [17] proposed the concept that the fluid exchange between CSF and the inner ear may allow proinflammatory cytokines, chemokines, and immune cells within the CSF to enter and damage the inner ear. Our study supports this concept by demonstrating that liposomes and CCR2^+^ immune cells accumulate both inside the cochlear aqueduct and on the inside wall of the scala tympani in EAE animals. Furthermore, E-cadherin staining suggests that arachnoid barrier layer ends around each cochlear nerve bundle where the nerve bundle pass through the bony labyrinth, which appears similar to the discontinuous arachnoid barrier around the olfactory nerve bundles when they pass through the cribriform plate. It is likely that CSF at these arachnoid barrier termination points can leak into the inner ear, because liposomes and CCR2^+^ cells can be found in the spiral ganglia.

Numerous studies using CSF tracers with electron and fluorescence microscopy have shown that the optic nerves carry all three meningeal layers as they exit the skull distally until the sclera and thus contain a SAS continuous with the cerebral basal SAS [45, 51]. Our data were consistent with these reports and demonstrated that the E-cadherin^+^ arachnoid sheath surrounded the nerve until its termination at the orbit. Despite this apparently continuous arachnoid barrier layer, there are conflicting data in the literature about whether a barrier is truly present at this location. An ultrastructural tracer study performed in the rabbit showed the presence of numerous small tortuous channels within the connective tissue where the SAS of the optic nerve terminates [22]. Ferritin infused into the ventricles was present in these channels and subsequently reached the intra-orbital connective tissue. A more recent electron microscopy study performed in cats found excavations of the SAS in the distal portion of the optic nerve, and contrast medium infused in the cisterna magna leaked from the SAS to reach the orbit [45]. In the current study, the liposomes infused into the lateral ventricle of mice seem to be confined in the SAS of the optic nerve and were not found in the intra-orbital connective tissue. However, during EAE, abundant CCR2^+^ immune cells were found in the intra-orbital connective tissue. At this stage, we cannot claim with any certainty that these CCR2^+^ cells had migrated from the SAS of optic nerve. Further studies are warranted to address this question and to investigate how CNS-derived cells can breach the arachnoid layer to reach the intra-orbital connective tissue.

The olfactory nerve has gained increasing interest in recent years since many studies in animals have identified it as a major pathway for lymphatic vessel-mediated CSF efflux [29, 33, 82, 86]. The arachnoid barrier layer is discontinuous (sometimes described as interrupted) above the cribriform plate where the olfactory nerve bundles pass through to reach the glomeruli [31, 73]. If convective CSF flow facilitates the distribution of immune cells that infiltrate into the SAS, we would expect to find accumulation of CCR2^+^ cells downstream at the CSF efflux pathway. Indeed, we observed abundant liposomes and CCR2^+^ cells in this region, supporting this hypothesis. Dural lymphatics and lymphatics around the olfactory nerve near the cribriform plate have been proposed to play an important role in immune cell trafficking into or out of the CNS during homeostasis and neuroinflammation [2, 31]. We observed IBA1^+^ macrophages inside the cribriform plate lymphatics in healthy mice and CCR2^+^ cells inside these lymphatics in EAE mice, confirming previous studies [30, 31]. Using the Prox1-GFP reporter mice, we did not identify lymphatic vessels around the trigeminal or cochlear nerve. Yet, the infiltrated CCR2^+^ cells seen at the EAE peak stage were significantly reduced at EAE chronic stage. The mechanisms underlying the resolution of CCR2^+^ cell from these locations deserve further investigation.

Taken as a whole, our study has identified that CSF plays an important role in the distribution and potential resolution of immune cell lesions along cranial nerves. Numerous studies using EAE animal models have suggested that the leptomeninges and SAS are an initial infiltration site of autoreactive T cells [2, 5, 43, 66]. A previous study [67] described the involvement of CSF-filled SAS cisterns as sites of immune cell homing and circulation within the CNS during the course of EAE. Our previous study showed that CSF flow toward the caudal spinal cord was significantly impaired before immune cell infiltration occurred into the SAS of spinal cord, further supporting the notion that the complexity of CSF circulation needs to be taken into account to fully understand immune cell trafficking into or out of the CNS.^83^

One limitation of our study is that liposome distribution was only assessed 2 h post-i.c.v infusion. This relatively short period of circulation of liposomes versus several days of infiltration of CCR2 ^+^ cells may explain why CCR2 ^+^ cells are seen at locations (e.g., intra-orbital connective tissue, parenchyma of the trigeminal nerve) where liposomes were not found. However, active migration mechanisms of immune cells may be involved and need future investigation. Moreover, the EAE model used in our study substantially differs from human disease in terms of the pathogenicity of autoimmune antibodies, i.e., anti-MOG demyelinating antibodies. Thus, despite the histopathological similarities evidenced between the EAE model and MOGAD in this study, the described model cannot be utilized to elucidate the development of antibody-mediated demyelinating pathology. In addition, to what extent the anatomical structures, e.g., glia barrier and arachnoid barrier around the human cranial nerves, are similar to those of mice also needs further investigation. It may be feasible to anatomically assess the arachnoid barrier and glia cell distribution along optic and trigeminal nerves in human postmortem tissues, while the human cochlear nerve transition zone may be studied on decalcified human tissues as previously described [65].

In summary, our study provides significant evidence that the MOG_35-55_-driven EAE model recapitulates the inflammatory lesions seen around cranial nerves in patients with inflammatory demyelinating disease. CSF tracer studies combined with immunofluorescence staining of markers for the glia and arachnoid barriers in EAE mice allowed us to elucidate the anatomical details and accessibility to CSF flow at the CNS–PNS TZ of trigeminal nerves and cochlear nerves. This knowledge will benefit not only continuing research on the pathogenesis of autoimmune demyelinating diseases but also potentially other conditions such as pathologies of the inner ear and the debilitating trigeminal neuralgia associated with other conditions, such as vascular compression. Furthermore, the potential involvement of CSF flow in disseminating CNS-derived immune cells to the inner ear and to the proximal segment of trigeminal nerve suggested by our results may encourage an underappreciated CSF-mediated delivery route of therapeutics to treat inner ear inflammation and trigeminal nerve inflammation.

## Supplementary Information

Below is the link to the electronic supplementary material.

Supplementary file1 (DOCX 10190 KB)

## Data Availability

All data generated or analyzed during this study are included in this article and its Supplementary Information files. All requests for raw data should be addressed to the corresponding authors.

## Abbreviations

MS: Multiple sclerosis
MOGAD: MOG-associated disease
TZ: CNS–PNS transition zone
CSF: Cerebrospinal fluid
CNS: Central nervous system
PNS: Peripheral nervous system
ON: Optic neuritis
NMOSD: Neuromyelitis optica spectrum disorder
REZ: Root entry zone
SAS: Subarachnoid space
i.c.v: Intracerebroventricular infusion
MOG: Myelin oligodendrocyte glycoprotein
PLP: Myelin proteolipid protein
MBP: Myelin basic protein
EAE: Experimental autoimmune encephalomyelitis
NFL: Nerve fiber layer
CFA: Complete Freund's adjuvant
PTX: Pertussis toxin
MOG_35-55_: Myelin oligodendrocyte glycoprotein peptide
EDTA: Ethylenediaminetetraacetic acid
GFAP: Glial fibrillary acidic protein
AQP: Aquaporin
