# Supplementary material for: Role of CSF flow and meningeal barriers in the development of inflammatory lesions at the CNS–PNS transition zone of cranial nerves in autoimmune demyelinating diseases
Source: Acta Neuropathol. 2025 Jun 19;149(1):65. doi: 10.1007/s00401-025-02896-1 (PMC12178981; doi:10.1007/s00401-025-02896-1)
Supplement: Supplementary file 1 — Supplementary file1 (DOCX 10190 KB) [file 401_2025_2896_MOESM1_ESM.docx]

**Supplemental figures**

**Role of CSF flow and meningeal barriers in the development of inflammatory lesions at the CNS-PNS transition zone of cranial nerves in autoimmune demyelinating diseases**

**Li Xin^1*^, Hideaki Nishihara^2^, Adrian Madarasz^1^, Petr Pleskac^1^, Linh Tran^1^, Daniela C. Ivan^1^, Fumitaka Shimizu^2^, Simone Aleandri^3^, Giuseppe Locatelli ^1^, Paola Luciani^3^, Steven T. Proulx^1*^**

^1^Theodor Kocher Institute, University of Bern, Bern, Switzerland

^2^ Department of Neurology and Clinical Neuroscience, Yamaguchi University, Yamaguchi, Japan

^3^ Department of Chemistry, Biochemistry and Pharmaceutical Sciences, University of Bern, Bern, Switzerland.

*Corresponding authors:

Li Xin, PhD

Theodor Kocher Institute

University of Bern

CH‑3012 Bern, Switzerland

Email: [li.xin@unibe.ch](mailto:li.xin@unibe.ch)

Steven T. Proulx, PhD

Theodor Kocher Institute

University of Bern

CH‑3012 Bern, Switzerland

Email: [steven.proulx@tki.unibe.chs](mailto:steven.proulx@tki.unibe.chs)


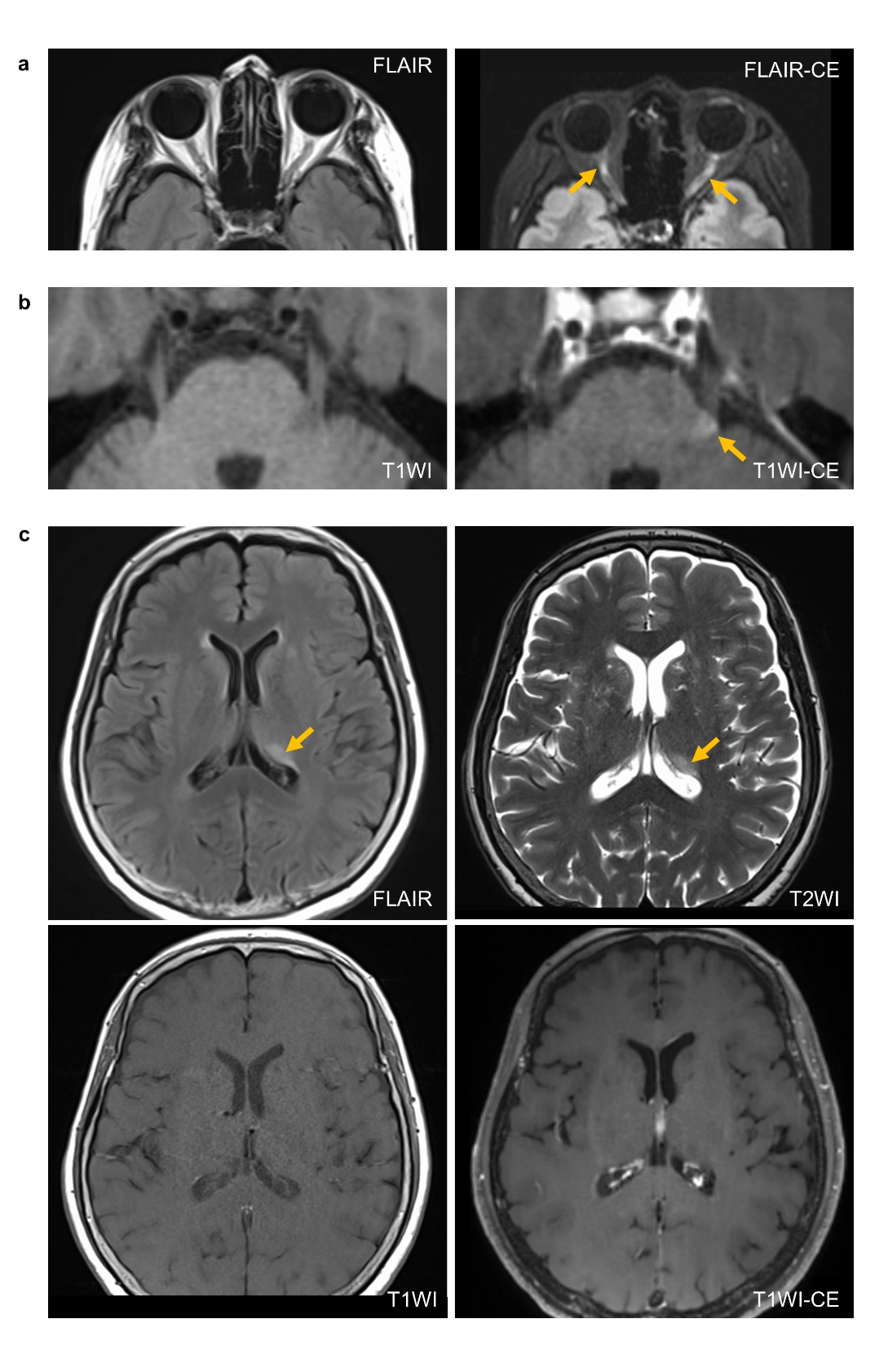


**Supplemental Fig. 1 Additional MRI imaging of case 1 (patient with an inflammatory demyelinating disease of undefined type) at acute phase.** Axial fluid-attenuated inversion recovery (FLAIR) and T1-weighted images (T2WI) revealed bilateral optic nerve lesions (**a**) and lesion in the left intramedullary trigeminal tract at REZ (**b**) exhibited contrast enhancement (T1W1-CE). In contrast, the periventricular hyperintense lesion seen on FLAIR and T2W1 in the acute phase did not show contrast enhancement. Yellow arrows indicate lesions.


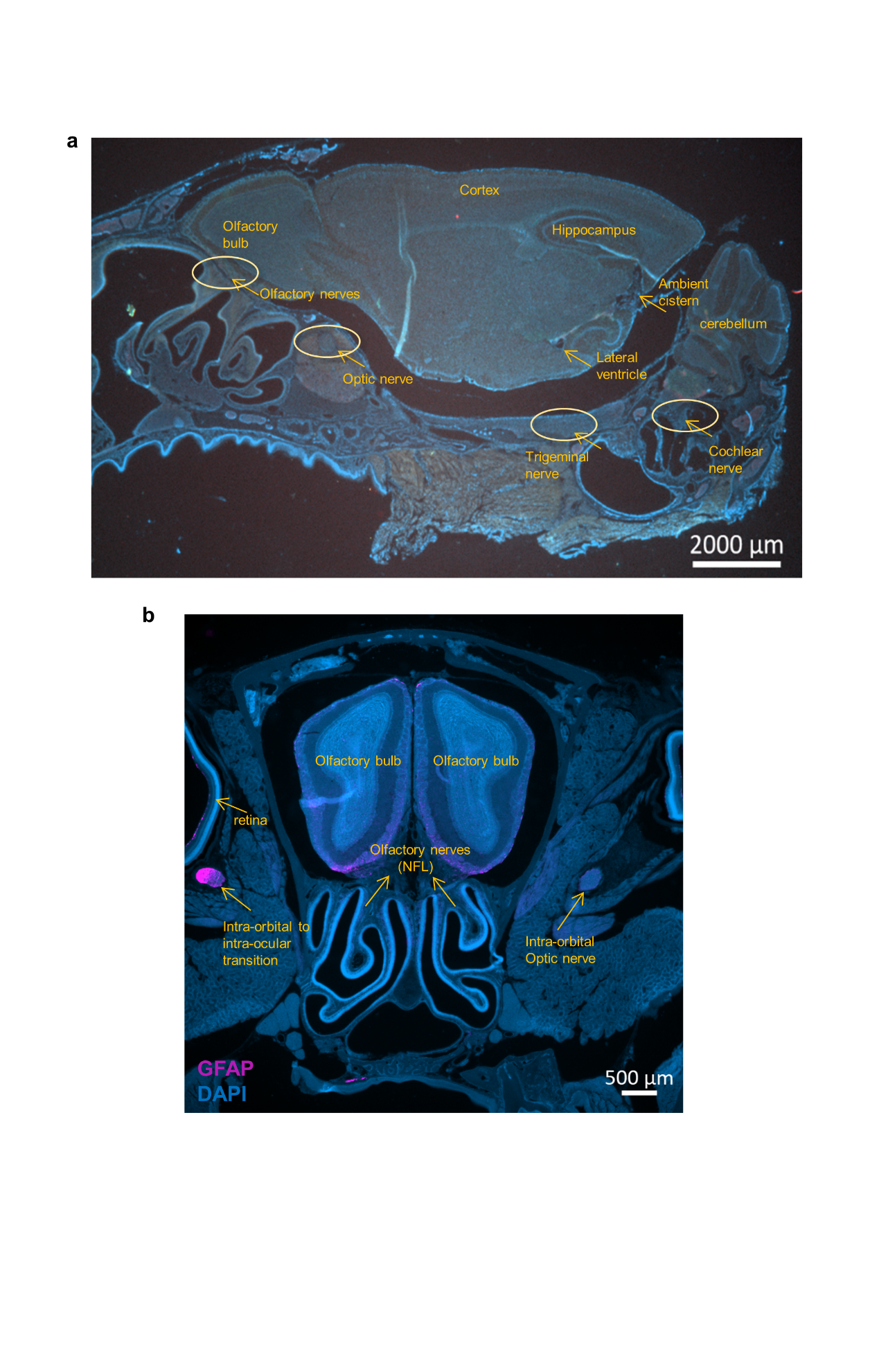


**Supplemental Fig. 2 Decalcified tissues marking the relevant anatomical landmarks. a** A sagittal section of the decalcified cranium showing the four cranial nerves assessed in this study and other brain structures indicated by the arrows. **b** A coronal section of the decalcified rostral cranium with GFAP staining showing the olfactory bulbs, olfactory nerve fiber layer (NFL) and intra-orbital segment of the optic nerve (right). The optic nerve transition area (from intra-orbital to intra-ocular) is indicated on the left, near the retina.


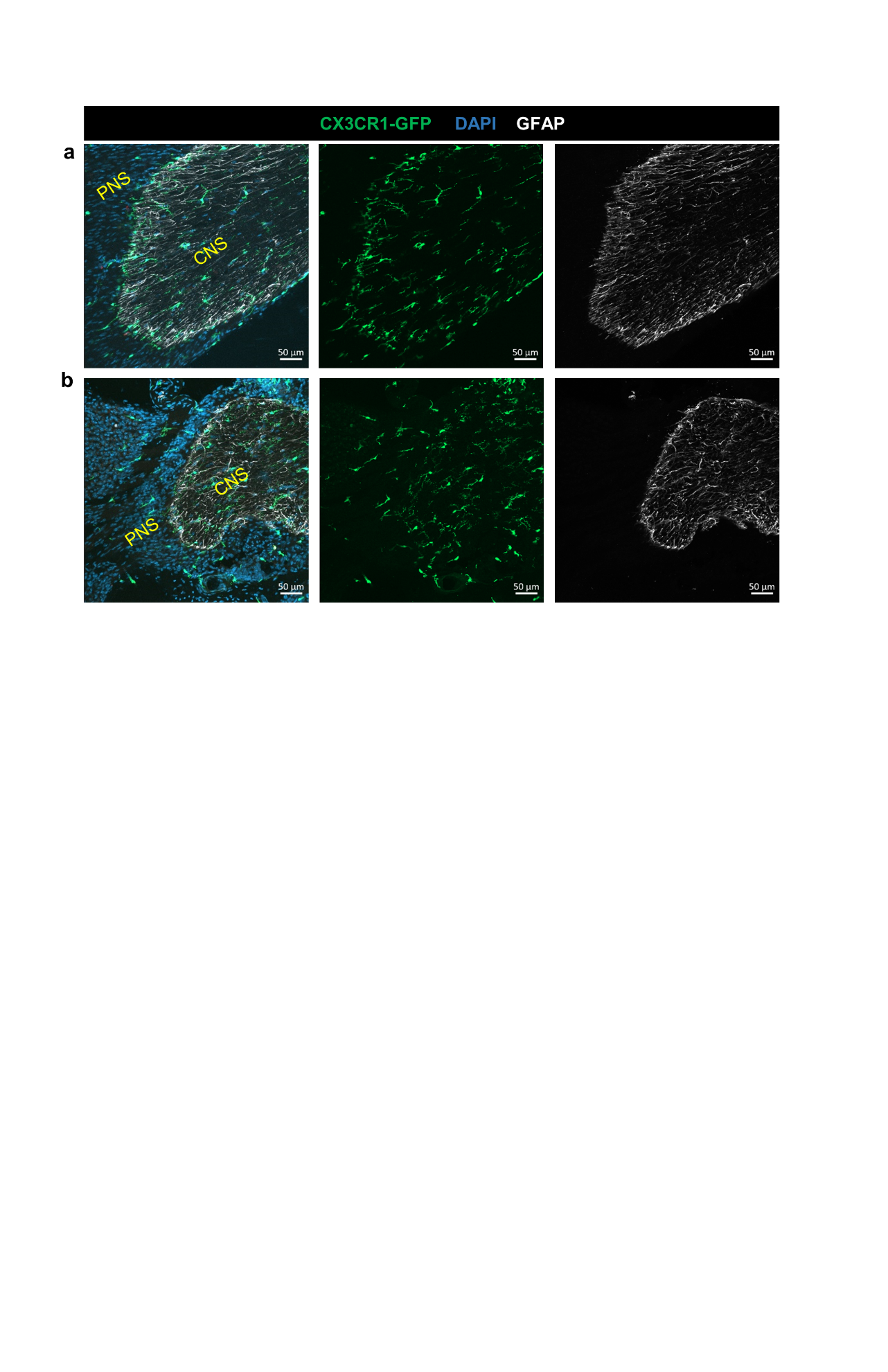


**Supplemental Fig. 3 CX3CR1^+^ cell distribution between the CNS and PNS segments of the trigeminal and cochlear nerve. a** CX3CR1^+^ microglia were abundant within the CNS segment of the trigeminal nerve and largely associated with the dome-shaped TZ marked by GFAP^+^ astrocyte foot processes. CX3CR1^+^ cells were scarce on the PNS side of the trigeminal nerve. **b** Cx3CR1^+^ cells were abundant on the CNS segment of the cochlear nerve and scarce on the PNS side, but their distribution pattern did not clearly reveal the TZ marked by GFAP^+^ astrocyte foot processes.


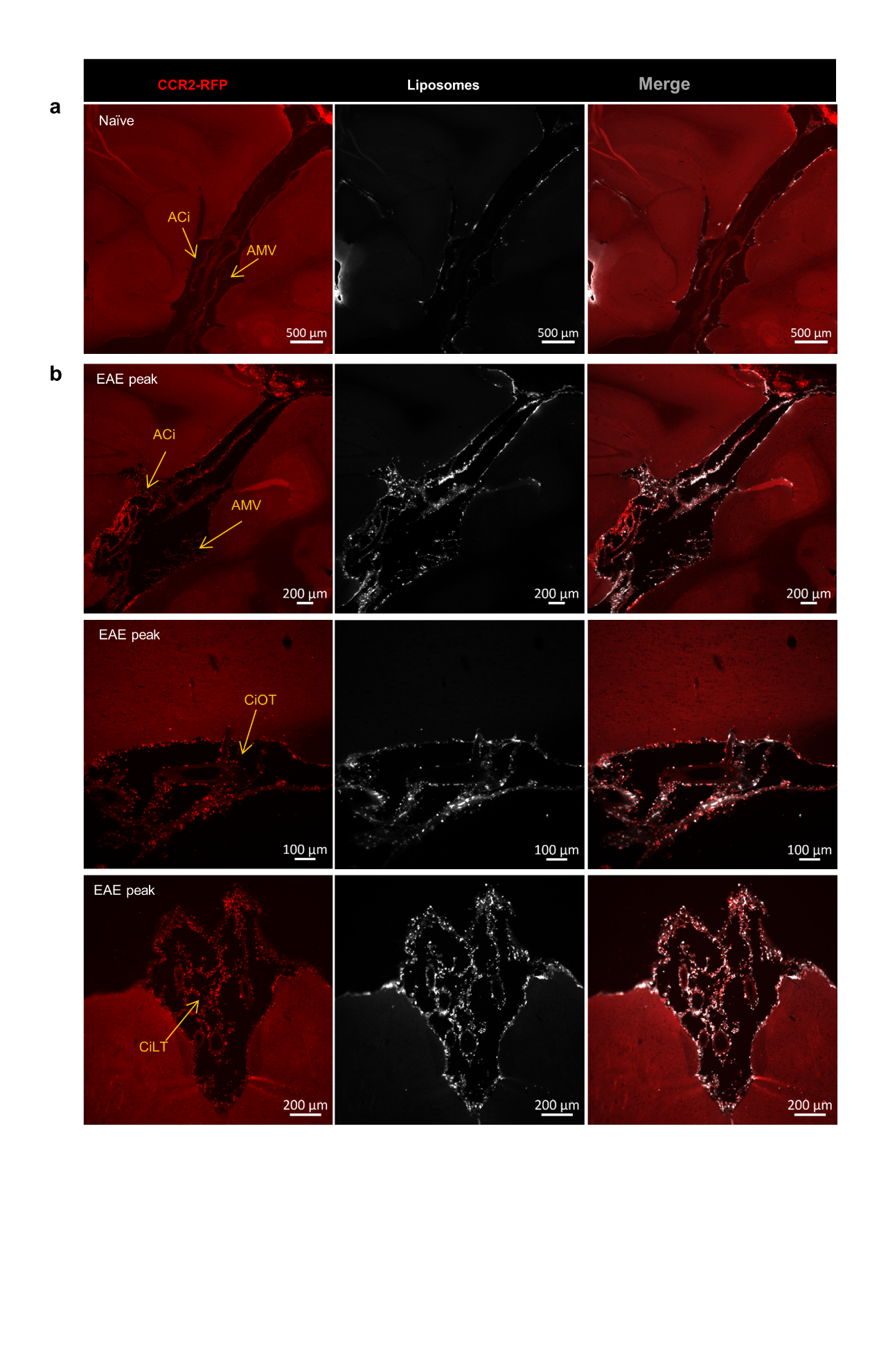


**Supplemental Fig. 4 CCR2^+^ cells and liposomes are accumulated within the SAS cisterns. a** Representative images showing that in the naïve mouse, liposomes were visible in the SAS cisterns, but CCR2^+^ cells were nearly visible. ACi, ambient cistern; AMV, anterior medullary velum. **b** In EAE peak stage, accumulation of CCR2^+^ cells and liposomes were seen in multiple SAS cisterns including the ACi, AMV, the CiOT (cistern of the optic tract), and the CiLT (cistern of the lamina terminalis).


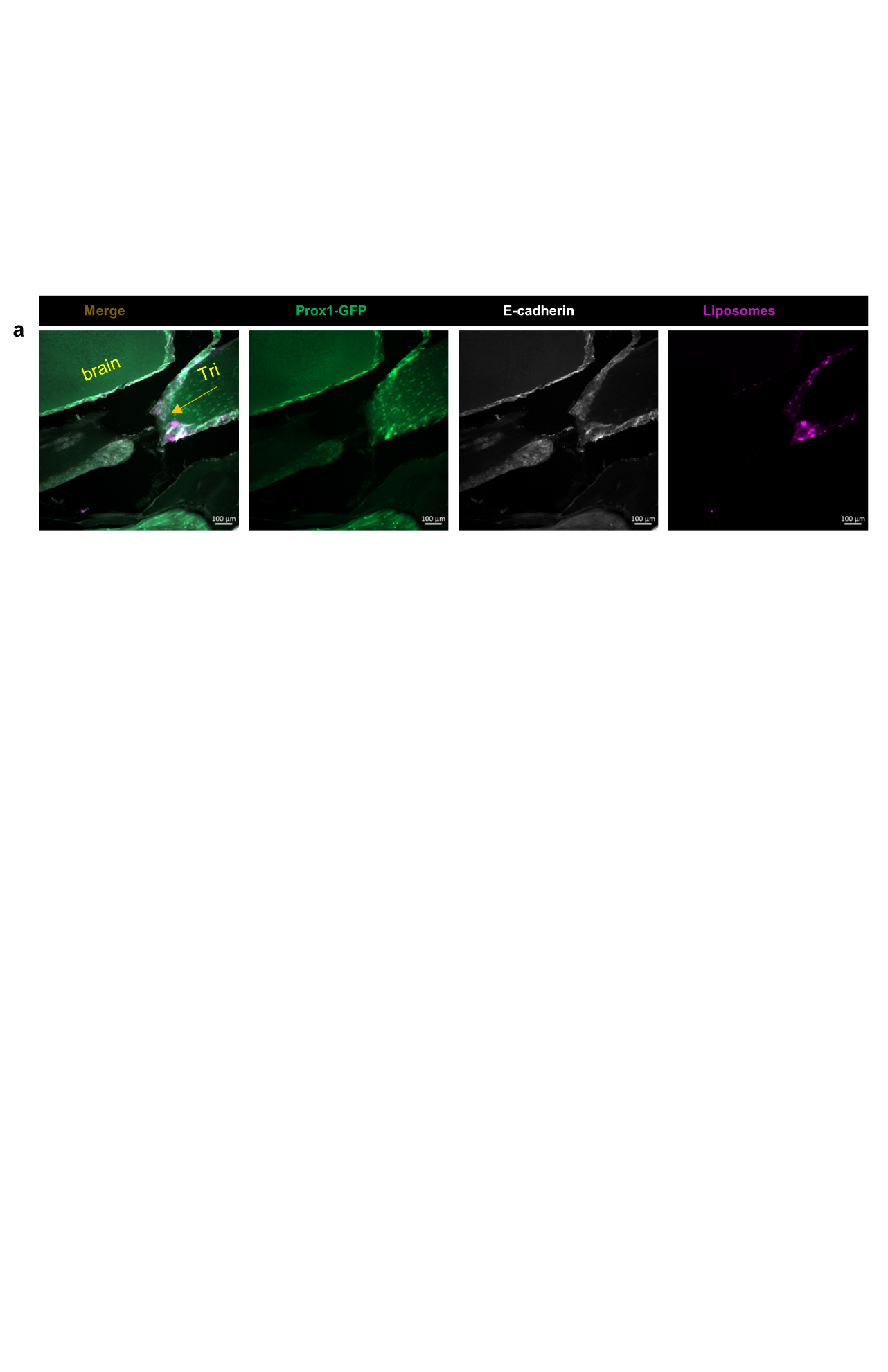


**Supplemental Fig. 5 Arachnoid cul-de-sac of the trigeminal nerve. a** The arachnoid cul-de-sac (arrow) of the trigeminal nerve (Tri) is revealed on a lateral sagittal section of the Prox1-GFP mouse i.c.v-infused with liposomes.


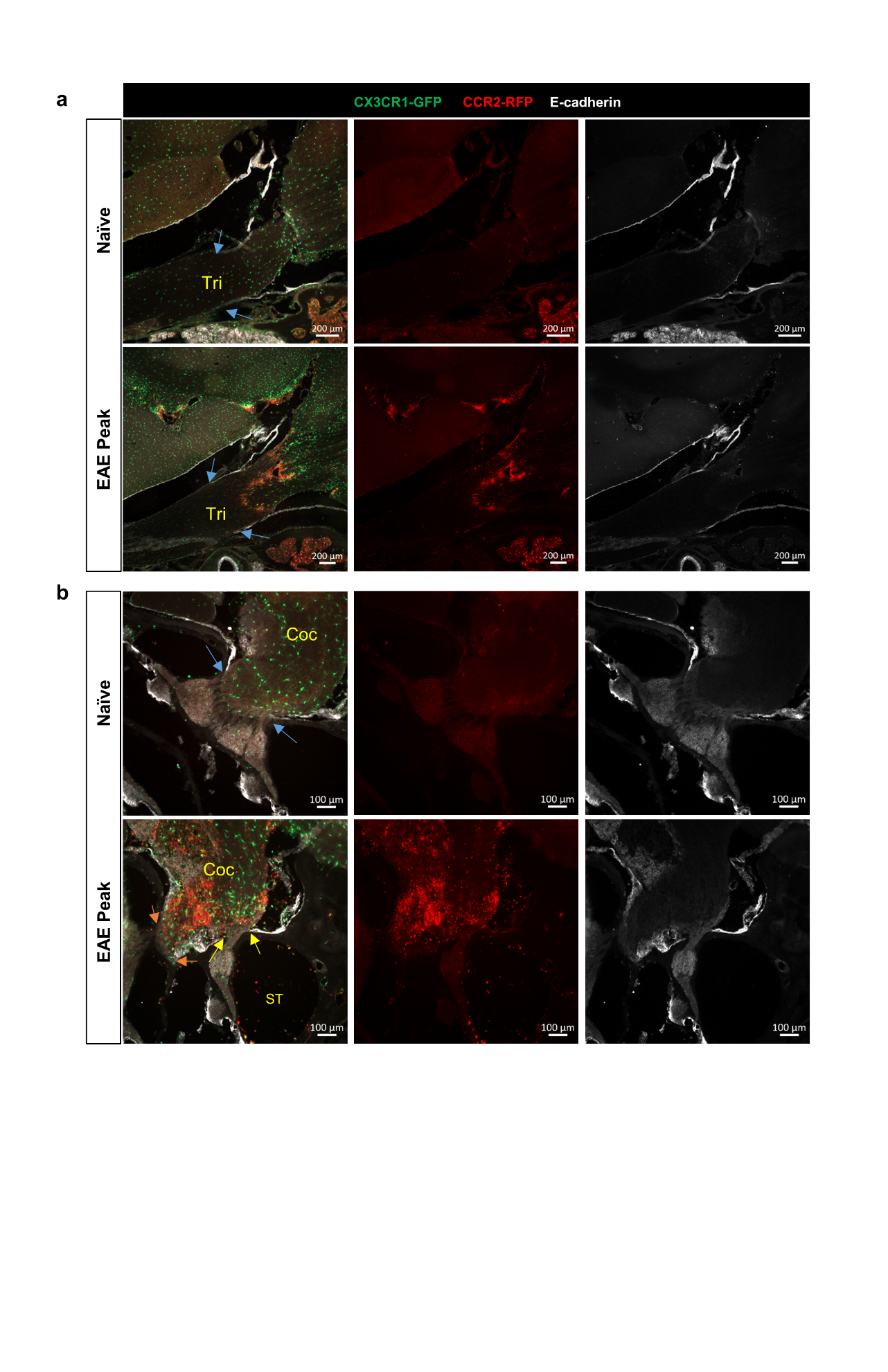


**Supplemental Fig. 6 E-cadherin staining did not reveal obvious differences between the naïve and EAE condition for trigeminal and cochlear nerves.** **a** E-cadherin staining did not reveal morphological differences around the trigeminal nerve (Tri) between the naïve and the EAE mouse ((n= 3 mice in each group). **b** Discontinuous E-cadherin^+^ signal (between the colored arrows) when nerve bundles merge into the trunk of cochlear nerve (Coc) was seen in both naïve and EAE peak. ST, scala tympani.


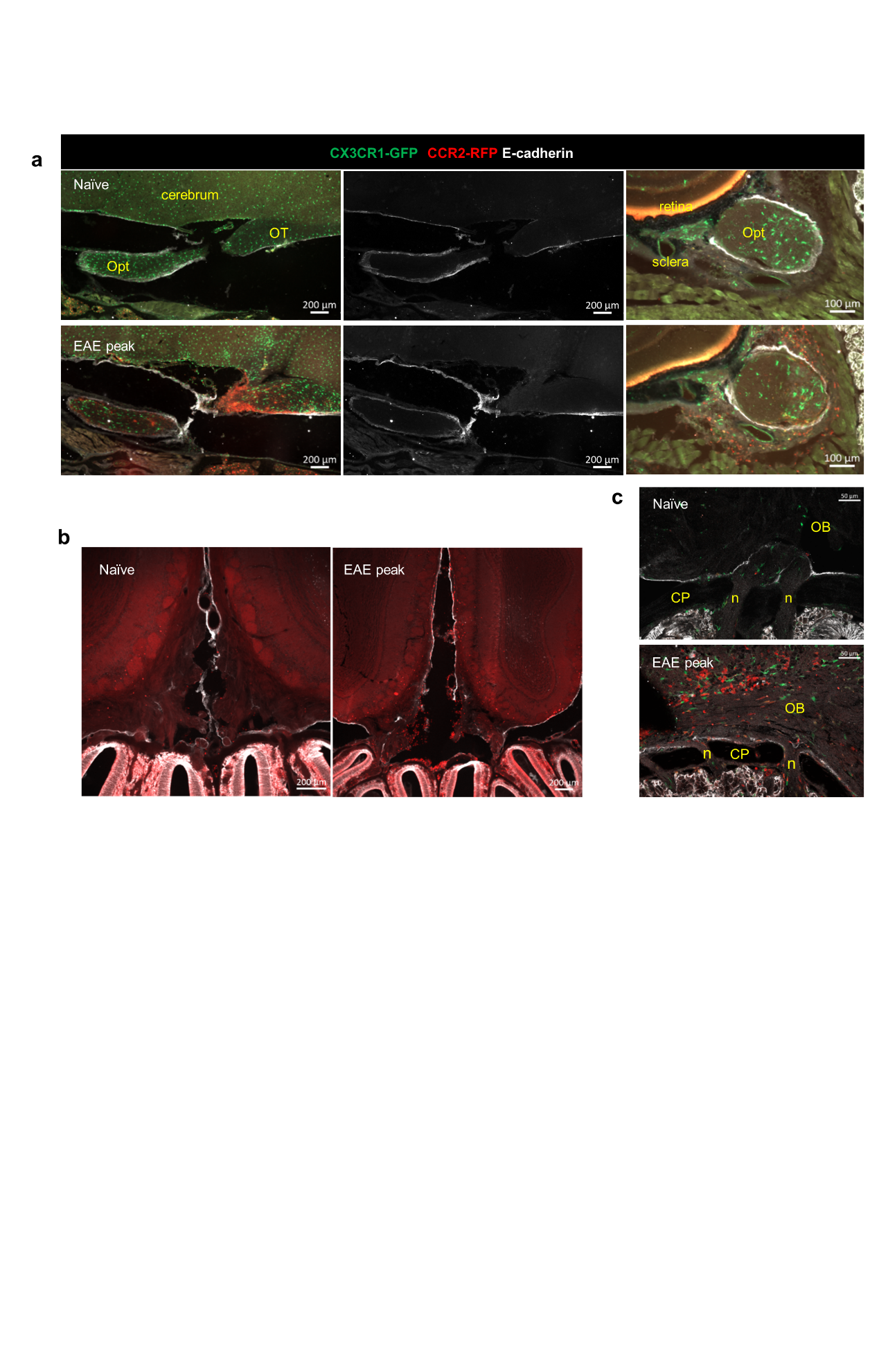


**Supplemental Fig. 7 E-cadherin staining did not reveal obvious differences between the naïve and EAE mouse for optic and olfactory nerves.** **a** E-cadherin staining on sagittal sections (left and middle panels) of the optic tract (OT) and the cranial segment of the optic nerve (Opt). Right panels, E-cadherin staining on coronal sections showing the optic nerve (Opt) near the retina. CCR2^+^ cells were visible inside the optic nerve parenchyma, as well as in the intra-orbital connective tissue. **b** The discontinuous E-cadherin staining looks similar around the olfactory nerve bundles between the naïve and EAE mouse. **c** Representative confocal images showing the indifferentiable E-cadherin staining pattern between the naïve and the EAE condition.


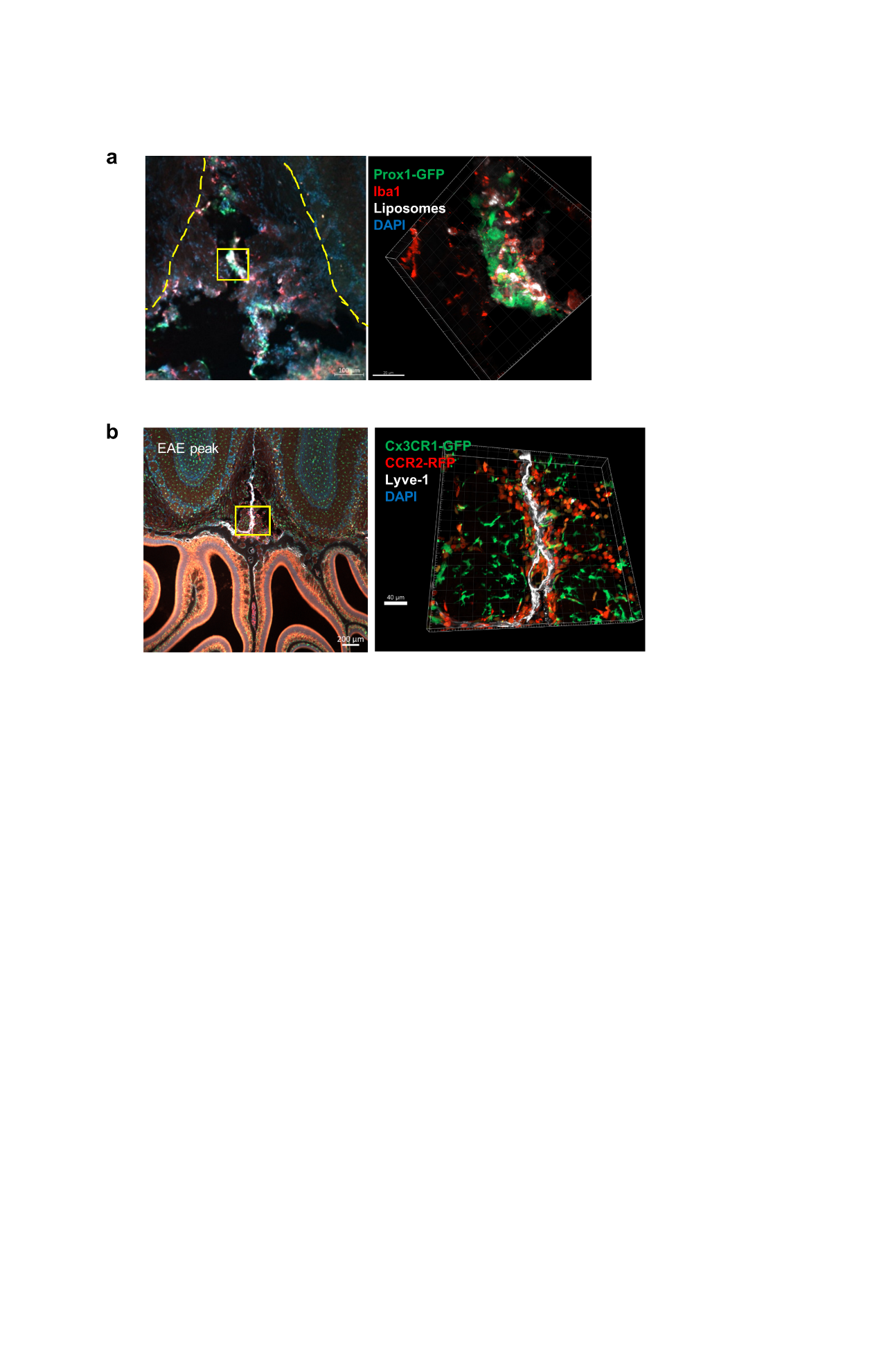


**Supplemental Fig. 8 Immune cells were found inside the cribriform plate lymphatic vessels in naïve and EAE mouse. a** Iba1^+^ macrophages containing liposomes observed inside the lymphatic vessel in a naïve Prox1-GFP mouse. Right panel, the 3D view of a confocal image from the yellow box area. **b** During EAE, CCR2^+^ cells were located within the lumen of the Lyve-1^+^ cribriform plate lymphatic vessels. Right panel, the 3D view of a confocal image from the yellow box area.
